# Dynamic interplay between the periplasmic chaperone SurA and the BAM complex in outer membrane protein folding

**DOI:** 10.1038/s42003-022-03502-w

**Published:** 2022-06-08

**Authors:** Bob Schiffrin, Jonathan M. Machin, Theodoros K. Karamanos, Anastasia Zhuravleva, David J. Brockwell, Sheena E. Radford, Antonio N. Calabrese

**Affiliations:** Astbury Centre for Structural Molecular Biology, School of Molecular and Cellular Biology, Faculty of Biological Sciences, University of Leeds, Leeds, LS2 9JT UK

**Keywords:** Structural biology, Membrane structure and assembly

## Abstract

Correct folding of outer membrane proteins (OMPs) into the outer membrane of Gram-negative bacteria depends on delivery of unfolded OMPs to the β-barrel assembly machinery (BAM). How unfolded substrates are presented to BAM remains elusive, but the major OMP chaperone SurA is proposed to play a key role. Here, we have used hydrogen deuterium exchange mass spectrometry (HDX-MS), crosslinking, in vitro folding and binding assays and computational modelling to show that the core domain of SurA and one of its two PPIase domains are key to the SurA-BAM interaction and are required for maximal catalysis of OMP folding. We reveal that binding causes changes in BAM and SurA conformation and/or dynamics distal to the sites of binding, including at the BamA β1-β16 seam. We propose a model for OMP biogenesis in which SurA plays a crucial role in OMP delivery and primes BAM to accept substrates for folding.

## Abstract (web summary)

Interaction of the outer membrane protein (OMP) chaperone SurA and the OMP folding catalyst BAM results in changes in the conformational ensembles of both species, suggesting a mechanism for delivery of OMPs to BAM in Gram-negative bacteria.

## Introduction

**Fig. 1:**
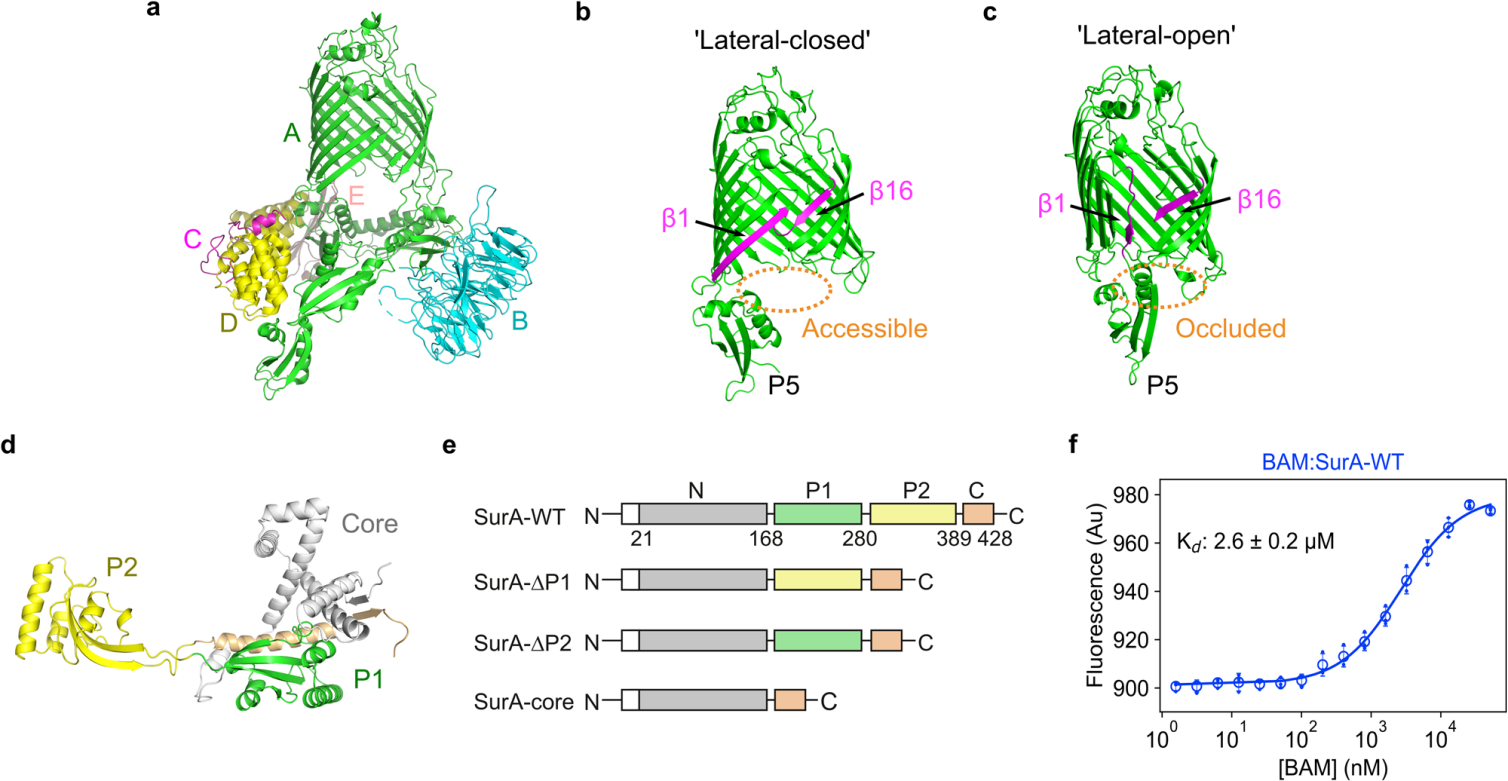
Structure and interaction of SurA and BAM. **a** Structure of BAM in the lateral-closed state (PDB: 5D0O^11^). Subunits are coloured BamA (green), BamB (cyan), BamC (pink), BamD (yellow), BamE (light pink). **b**, **c** Structures of the BamA β-barrel domain and POTRA 5 (P5) from BAM structures in **c** the lateral-closed (‘inward-open’) state (PDB: 5D0O^11^), and **d** the ‘lateral-open’ state (PDB: 5LJO^13^). β-strands 1 and 16 of the BamA β-barrel, which form the lateral gate, are highlighted in magenta. The entrance to the BamA β-barrel lumen is accessible in the ‘lateral-closed’ conformation, but is occluded in the ‘lateral-open’ BAM conformation, as indicated in orange. **d** Crystal structure of *E. coli* SurA (PDB: 1M5Y^28^). Regions are coloured grey (N-terminal region of the core domain), green (P1), yellow (P2) and orange (C-terminal region of the core domain). The colour scheme for BAM subunits and SurA domains is used throughout. **e** Domain architecture of *E. coli* SurA-WT and the SurA domain deletion variants used in this study. The signal sequence is not shown and was not present in any of the constructs used here, but the numbering used throughout reflects the gene numbering (including the signal peptide). Constructs were expressed with an N-terminal His_6_-tag and TEV cleavage site (white box). **f** Microscale thermophoresis (MST) data for binding of SurA-WT to BAM. Samples contained 400 nM Alexa Fluor 488-labelled SurA, BAM (1.6 nM–52 μM), 0.02% (v/v) DDM, 150 mM NaCl, 20 mM Tris–HCl, pH 8, at 25 °C. Three independent replicates were performed and averaged prior to fitting. The mean for each BAM concentration is shown as open circles and the individual values for each replicate are shown as dots. The error bars represent the standard deviation between replicates. Data were fitted to a 1:1 quadratic binding model (see the “Methods” section). Source data are provided as a Source Data file (Supplementary Data 8).

The outer membrane (OM) of Gram-negative bacteria is densely packed with outer membrane proteins (OMPs) that perform a range of functions essential for cell survival and virulence^1–3^. OMP biogenesis involves multiple handover events of unfolded OMP clients between folding factors spanning the cytoplasm, inner membrane, periplasm and outer membrane, including the cytoplasmic chaperone SecB, the Sec translocon, periplasmic chaperones (e.g. Skp, SurA. FkpA and DegP) and the β-barrel assembly machinery (BAM), which resides in the OM and acts as the terminal OMP foldase^4–6^. *E. coli* BAM is a ∼203 kDa heteropentameric complex which is comprised of the major conserved subunit BamA, itself an OMP, and four accessory lipoproteins BamB–E (Fig. 1a). *E. coli* BamA consists of a 16-stranded transmembrane β-barrel domain and a periplasmic region comprising five polypeptide-transport-associated (POTRA) domains. BamA and BamD are essential for *E. coli* viability^7,8^, with BamB mutants exhibiting the greatest OMP assembly defects of the non-essential lipoproteins^9^. The BamA β-barrel contains a lateral-gate at the interface between β-strands 1 and 16, and structural data have captured this gate in both lateral-closed and lateral-open states^10–13^ (Fig. 1b, c). The potential opening and closing motions of the lateral gate are accompanied by movements of the POTRA domains^10–13^. In the lateral-open state POTRA 5 occludes access to the barrel lumen from the periplasmic face of BAM, whereas in the lateral-closed conformation (also known as ‘inward-open’) POTRA 5 is positioned away from the barrel lumen, potentially providing access for the incoming OMP to the BamA β-barrel (Fig. 1b, c).

SurA is considered the major OMP chaperone in the periplasm^14–19^ and evidence suggests that it has the primary responsibility for OMP delivery to BAM^14,15,20–22^. SurA deletion in a number of species has been shown to result in OM assembly defects (including impaired assembly of virulence factors, e.g. pili and adhesins)^23–25^, reduced pathogenicity^23–25^, the induction of stress responses, and a loss of OM integrity, as measured by increased sensitivity to antibiotics and detergents^16,18,23,26,27^. *E. coli* SurA is comprised of three domains: a core domain, made of its N- and C-terminal regions, and two parvulin-like peptidylprolyl isomerase (PPIase) domains (P1 and P2) (Fig. 1d, e)^28^. Despite the availability of high-resolution structural data from X-ray crystallography^28,29^, mechanistic details of how SurA chaperones its OMP clients and delivers them to BAM, and how this is choreographed and controlled in the absence of ATP, have remained obscure^5^. Recent in vitro evidence has shed light on the mechanism of substrate interaction by SurA, and revealed that (1) the core domain of SurA contains the primary OMP binding sites^30,31^, (2) OMPs bind in a cradle formed between the core and P1 domains^30,31^, (3) the chaperone has a malleable architecture that responds to client binding^30,32,33^, and (4) clients bound to SurA can populate extended states^31,34,35^. However, despite this wealth of information, little is known about how SurA delivers its OMP clients to BAM to promote their folding. Folding studies in vitro using a reconstituted BAM in proteoliposomes and the aggregation-prone substrate OmpT showed that increases in SurA concentration lead to increased OmpT activity, as monitored by cleavage of a fluorogenic peptide^36,37^. However, it was unclear if this effect resulted from SurA reducing the misfolding and/or aggregation of OmpT, or reflected a role for SurA in directly increasing the rate of OMP folding and membrane insertion via BAM. A subsequent study on the BAM-mediated folding of BamA showed that SurA can be functionally replaced by 0.8 M urea^38^, suggesting that the chaperone may function as a solubility buffer for OMPs in the periplasm. Further, the presence of SurA reduced the observed folding rate of OmpA in BAM-containing proteoliposomes composed of 1-palmitoyl-2-oleoyl-sn-phosphatidylcholine (POPC)^39^. However, as OmpA can fold efficiently into POPC liposomes in the absence of BAM^39,40^, it is possible that the reduction in observed folding rate is due to SurA holdase activity inhibiting the unassisted folding pathway.

How SurA and the other OMP folding factors work together to coordinate OMP assembly, and the precise mechanism by which BAM folds and inserts OMPs (that have a wide range of sizes and structural complexity^41^) into the OM are still unclear^35,42–45^. In vitro and in silico evidence point to a role for BAM in membrane thinning and disordering of the OM bilayer to aid OMP folding^46–50^. Models in which the substrate makes direct interactions with the BamA barrel have also been proposed^5,6,45,51–53^, and are supported by a variety of experimental evidence^54–56^, including in vivo crosslinking^53,57,58^ and structural data^59–62^. For example, a cryoEM structure of a late-stage folding intermediate of BamA being folded by BAM^59^, as well as recent structures of EspP stalled while folding on BAM^61,62^, indicate that the terminal strand of an incoming OMP makes a β-augmentation interaction with the β1 strand of the BamA barrel as part of the assembly mechanism. Additional structures of BamA/BAM in which OMP-derived β-strands^60,63^ or darobactin (a peptide antibiotic that targets BamA)^64,65^, are bound to β1 of the BamA barrel suggest that the lateral-closed state may be responsible for the receipt of unfolded OMPs for folding into the OM. However, the initial interactions that mediate OMP recognition by BAM are undefined, as are the mechanistic roles that the periplasmic chaperones play in OMP delivery to BAM. In vivo crosslinking suggests that there is a direct interaction between SurA and BAM^14,22,66^, and a supercomplex spanning the periplasm including BAM, SurA, and the SecY holotranslocon has also been proposed as a folding conduit for OMPs^67–69^.

Here, we have exploited the sensitivity of differential hydrogen-exchange mass spectrometry (HDX-MS)^70–72^ to map regions mediating the interaction between BAM and SurA. Specifically, we identify three primary binding regions in BAM that include residues in: (1) POTRA domains 1 and 2 of BamA; (2) BamB; and (3) BamE, and reveal that the SurA P2 domain as well as its core domain are involved in BAM binding. Consistent with this, in vitro folding assays showed that SurA accelerates BAM-assisted folding of the transmembrane domain of OmpA (tOmpA), and that the SurA P2 domain is required for maximal acceleration. Changes in deuterium uptake in the BamA β-barrel upon SurA binding were also observed using HDX-MS, indicating that interaction with SurA modulates the structure and/or dynamics of BAM distal to the SurA binding site. Inspired by recent advances in computational protein structure prediction^73–75^ we used AlphaFold to generate a model of the SurA–BAM complex and show that this is consistent with the HDX data we present and previous literature reports^9,14,20,22,27,42,66,69,76,77^. Examination of this model also shows that further dynamic interconversions/alternative structures are required to explain all our results. Combined, our data support a model in which SurA directly facilitates OMP folding by targeted delivery of substrates to BAM and, by SurA-driven conformational changes, primes BAM to perform its catalytic function of efficiently folding OMPs into the OM.

## Results

### The core and P2 domains of SurA directly contact BAM

**Fig. 2:**
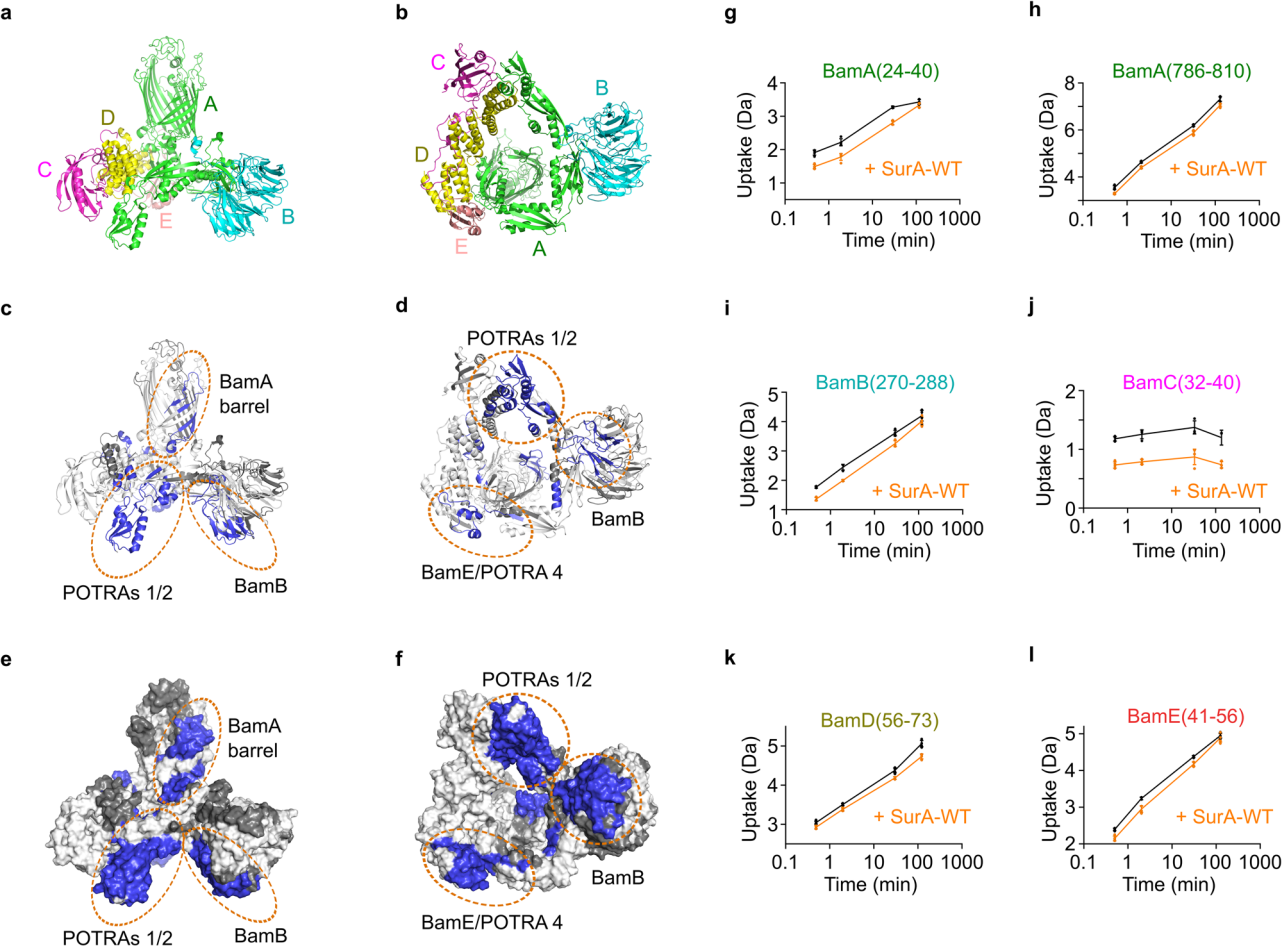
HDX-MS analysis of BAM in the presence of SurA reveals multiple chaperone binding sites and allosteric conformational changes. **a**, **b** The structure of the BAM complex in the ‘lateral-open’ conformation (PDB: 5LJO^13^) coloured by subunit. **c**, **d** Cartoon views and **e**, **f** surface views showing regions of HDX protection in the BAM complex upon binding SurA. Left panels show a side view of the complex, and right panels a view from the periplasmic face. Regions that are protected from hydrogen exchange in the presence of SurA are highlighted in blue. Regions in white show no change in deuterium uptake in the presence of SurA, while those in dark grey denote sequences for which peptides were not detected. Note that no regions showing deprotection from HDX upon SurA binding were observed. Patches of protection from HDX upon SurA binding in the BamA β-barrel domain (adjacent to the lateral gate), POTRAs 1 and 2, BamB, and BamE/POTRA 4 are ringed in orange. **g**–**l** Representative deuterium uptake plots for peptides from **g**, **h** BamA, **i** BamB, **j** BamC, **k** BamD, and **l** BamE. The extent of deuterium uptake (Da) in the absence (black) and presence of SurA (orange) is shown. Individual data points for each time point are shown as dots and error bars represent the standard deviation of three technical replicates. Source data are provided as a Source Data file (Supplementary Data 9).

To demonstrate whether a direct physical interaction between SurA and the BAM complex could be detected in vitro we first measured the affinity between SurA and BAM by microscale thermophoresis (MST). Using SurA labelled with Alexa Fluor 488 via an N-terminal cysteine residue, and purified BAM in DDM micelles^78^, clear evidence for a direct 1:1 interaction was observed, with a *K*_d_ of 2.6 ± 0.2 µM (Fig. 1f). Next, to obtain insights into the architecture of the SurA–BAM complex we employed differential hydrogen–deuterium exchange-mass spectrometry (HDX–MS) experiments to determine regions in BAM that are protected/deprotected from exchange in the presence of wild-type SurA (SurA-WT). To ensure complex formation we used concentrations of BAM and SurA of 8 and 10 µM, respectively. The concentrations chosen allow examination of protection/deprotection of both BAM and SurA under conditions in which a comparable percentage bound (~60%) of both is achieved, and minimise the known potential for self-association of SurA^29,32^ and BAM^22^ which would complicate the data analysis. Sequence coverage of 81% was obtained for BamA and 71–99% for the BAM lipoproteins (Figs. 2 and S1). Peptides with significantly different deuterium uptake values were observed in all five BAM subunits in the presence of SurA-WT, consistent with BAM–SurA complex formation (Figs. S2, S3, S4, S5). Most of the peptides showing protection from deuterium exchange in the presence of SurA are located in BamA (14 peptides) and BamB (8 peptides), with smaller numbers of peptides located in BamC (2 peptides), BamD (2 peptides) and BamE (3 peptides). The largest differences in deuterium uptake (~8%) were in BamA and BamB (Figs. S2, S3), consistent with one or both of these subunits being the primary site(s) for SurA binding.

Visualised on the structure of BAM, the peptides that exhibited protection are clustered in distinct regions (Figs. 2, S4, and S5). Three main clusters of protection are discernible in the periplasmic region of BAM: one located in POTRA domains 1 and 2 of BamA (residues 21–98, 111–132), one on the side of the BamB β-propeller facing into the periplasmic POTRA ring (residues 189–289), and a third involving BamE (residues 39–72) and BamA POTRA 4 (residues 340–347) (Fig. 2, Supplementary Video 1, Supplementary Data 1). Additional small areas of protection were observed on BamA POTRA 3 facing into the periplasmic ring (residues 223–231), the ‘lasso-like’ N-terminal region of BamC^79^ (residues 32–40) and the 3_10_ helix of BamD which makes contact with the membrane (residues 123–132)^13,50^ (Figs. S4, S5). Strikingly, protection was also observed deep into the transmembrane β-barrel domain of BamA, specifically around β15–β16 and β12–β13 (residues 717–738, 786–808) (Fig. 2). Interestingly, these protected regions comprise, or are adjacent to, the barrel seam which plays a crucial role in OMP folding^5,45,52,59,65,80,81^), suggesting that BAM undergoes allosteric conformational changes upon SurA binding.

**Fig. 3:**
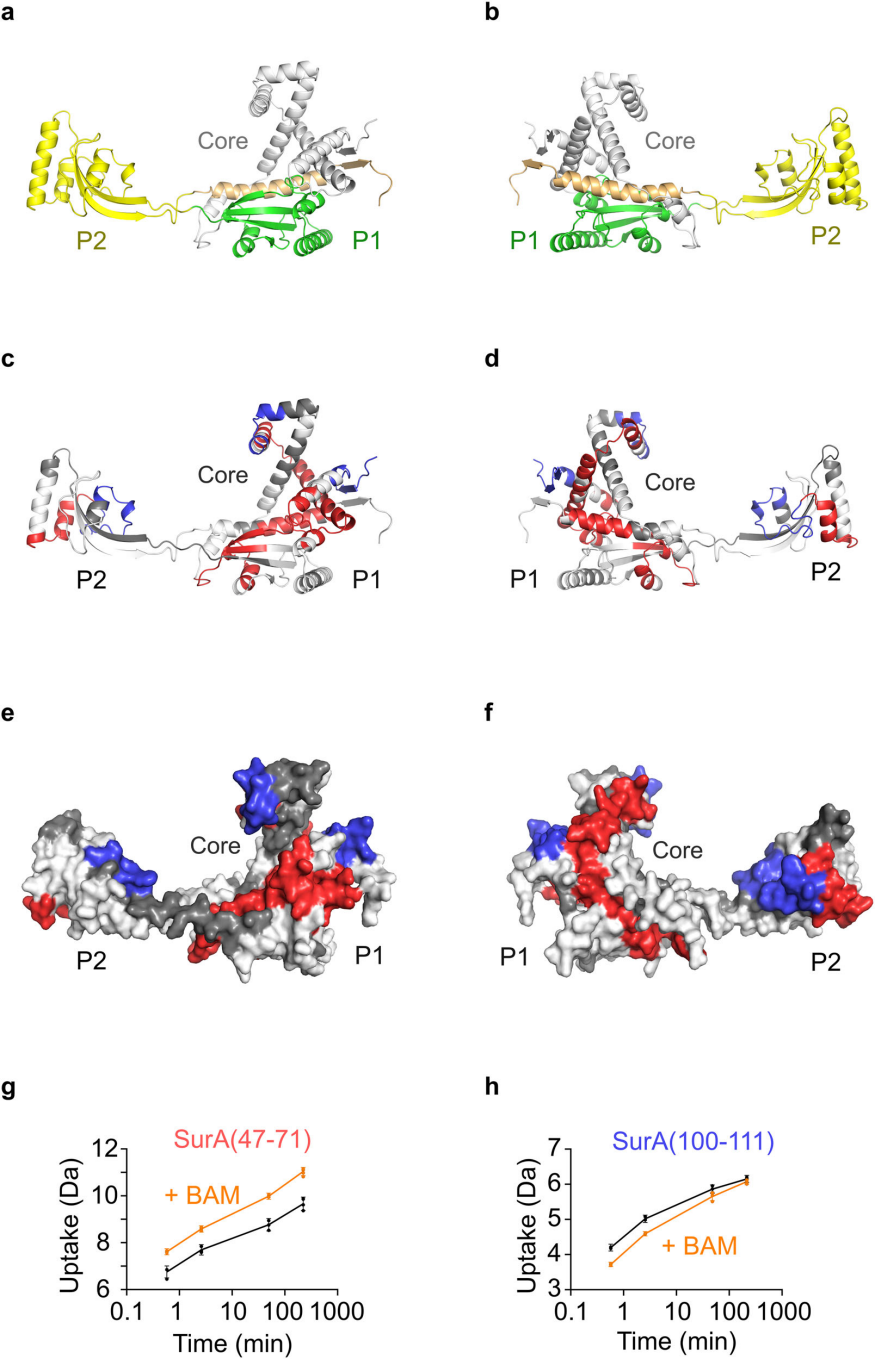
HDX-MS analysis of SurA in the presence of BAM reveals conformational changes in the chaperone upon binding. **a**, **b** The crystal structure of SurA (PDB: 1M5Y^28^) with the N-terminal and C-terminal regions of the core domain coloured in grey and orange, respectively. The SurA PPIase domains P1 and P2 are coloured in green and yellow, respectively. **c**, **d** Cartoon views and **e**, **f** surface views of differential HDX data for SurA upon binding BAM. Left panels show one view of the chaperone, and right panels a view rotated by 180° around the *y*-axis. Regions that are protected or deprotected from hydrogen exchange in the presence of BAM are highlighted in blue or red, respectively. Regions in white show no change in deuterium uptake in the presence of BAM, while those in dark grey denote sequences for which peptides were not detected. **g**, **h** Representative deuterium uptake plots for peptides spanning residues **g** 47–71 (deprotected) and **h** 100–111 (protected) from the core domain of SurA. The extent of deuterium uptake (Da) in the absence (black) and presence of BAM (orange) is shown. In the presence of BAM, these regions are deprotected and protected from exchange, respectively. Individual data points for each time point are shown as dots and error bars represent the standard deviation of three technical replicates. Source data are provided as a Source Data file (Supplementary Data 9).

Next, we studied the effect of BAM binding on SurA-WT by HDX-MS (Fig. 3). Protected peptides localise to three patches on SurA, two on opposite sides of the core domain, and one in the P2 domain (residues 21–38, 100–111, 328–350) (Figs. 3, S6, Supplementary Video 2, Supplementary Data 2). No protection was observed in the SurA P1 domain, suggesting either that P1 does not directly contact BAM, or that regions of P1 which interact with BAM also contact other SurA domains in the apo state and hence no net protection from exchange is observed in the complex with BAM. Notably, no regions of protection were observed within the cradle formed between the core and P1 domains of SurA, which previous data has implicated as the main OMP binding site^30,31^, suggesting distinct binding sites on SurA for OMPs and BAM. Intriguingly, several regions of deprotection were also observed when SurA binds BAM (Figs. 3 and S6). Deprotection was observed at the core–P1 interface (residues 212–243, 403–414), consistent with the opening of this interface observed previously using HDX-MS and single molecule FRET when SurA binds its clients OmpX and OmpF, or a P1-binding peptide (WEYIPNV)^30^. Deprotection was also observed across the SurA core (residues 47–96) and in the P2 domain (residues 311–326) (Figs. 3 and S6), indicating that additional conformational changes occur upon binding BAM. Together the results suggest that the PPIase domains of SurA spend less time associated with the core domain when SurA binds BAM, which may be important in the release of substrates from the binding cradle on engagement of SurA–OMP complexes with BAM.

Previous results have shown that the SurA core domain can functionally complement SurA-WT in vivo and in vitro, at least for some substrates^27,82,83^. We therefore also performed differential HDX-MS experiments in the presence of a SurA variant in which both PPIase domains had been removed (SurA-core). Binding experiments of SurA-WT and SurA-core to BAM in DDM showed that the proteins bind BAM with a similar *K*_d_ (2.6 ± 0.2 and 1.3 ± 0.3 µM, respectively), enabling direct comparison between the two pairs of experiments (Figs. 1f and S7). The differential HDX-labelling experiments showed that the region of protection within the BAM periplasmic ring clustered around BamE/POTRA4 observed with SurA-WT was no longer present in the presence of SurA-core. These data, combined with those from chemical crosslinking and functional data (see below), suggest that it is the P2 domain that makes contact with this region (Figs. S8 and S9). By contrast, the regions of protection located in BamA POTRA domains 1/2 and BamB remained, suggesting that these two areas comprise regions that bind SurA-core (Fig. S9). Interestingly, the regions of protection observed in the BamA barrel in the presence of SurA-WT were not observed in the presence of SurA-core. Therefore, while SurA-core can bind and deliver client proteins to BamA^27,83^, the same conformational changes are not relayed through the complex upon engagement of the chaperone, at least not to the same extent (Fig. S9).

Next, we performed chemical crosslinking-MS (XL-MS) to further investigate the key determinants of the BAM–SurA interaction (Fig. S10, Table S1). We used the homobifunctional NHS-ester disuccinimydyl dibutyric urea (DSBU) which crosslinks predominantly lysine residues within ca. 27 Å (Cα–Cα Euclidean distance) of each other^84^. We detected crosslinks from the P2 domain of SurA to BamE (residues K293 and K306 of SurA and K45 of BamE), BamA POTRA domains 3 and 4 (residues K293 and K306 of SurA and residues K216 and K309 of BamA), and BamD (residues K306 and K394 of SurA and residue K149 of BamD) (Fig. S10a, b), in agreement with the HDX-MS data (Figs. 2,  3, S2–5). Additionally, crosslinks from the SurA core domain were detected to BamB (residue K280), BamC (residues K166, K186, K261) and BamA POTRA 1 (residue K27) (Fig. S10a–c), in agreement with a SurA core binding site centred in this region of BAM, as identified by HDX-MS (Figs. 2 and S8). A further crosslink in the flexible linker between the P1 and P2 domains (residue K278) of SurA was observed to BamB (residue K280) (Fig. S10a), highlighting that the complex pattern of HDX-MS protection and deprotection observed for SurA in the presence of BAM does not preclude a P1–BAM interaction (Fig. 3).

Taken together, the binding, HDX-MS and cross-linking data provide the first molecular insights into how SurA binds BAM, demonstrating that this complex is weak (μM *K*_d_) and involves interactions between the BAM POTRA domains (specifically POTRAs 1, 2 and 4), BamB, and BamE, that interact predominantly with the core and P2 domains of SurA. In addition, changes in HDX protection in the BamA β-barrel upon SurA-WT binding (but not for SurA-core), combined with the regions of deprotection and protection observed in SurA upon BAM binding, suggest that SurA modulates the conformational dynamics of BAM and vice versa. This likely plays a role in priming both BAM and SurA for the delivery of unfolded OMPs from their chaperone-protected state to BAM for folding into the OM.

### The core and P2 domains of SurA enhance BAM catalysis of OMP folding

**Fig. 4:**
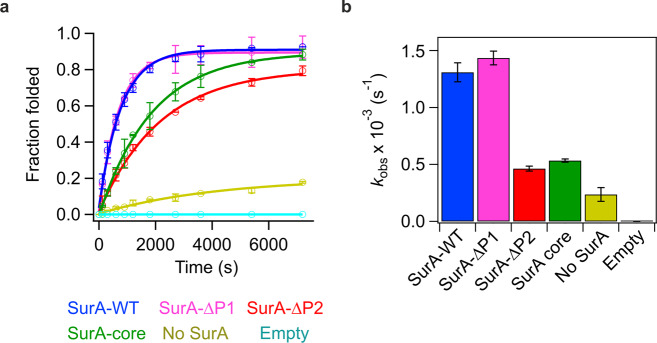
The presence of the SurA core and P2 domains are required for maximal rate enhancement of tOmpA folding on BAM. **a** Fraction folded of tOmpA in the presence or absence of BAM and SurA variants at different time points as measured by cold SDS–PAGE. Points are the mean of two independent replicates and error bars represent the range of values covered by the replicates. For BAM-containing samples fits to a single exponential equation are shown. In the absence of BAM no folding is observed. **b** Observed rate constants (*k*_obs_) from kinetic experiments in (**a**). Error bars represent the error on the fit to a single exponential. Example SDS–PAGE gels of the folding reactions are shown in Fig. S11. Samples contained 1 μM BAM (where included) in proteoliposomes containing *E. coli* polar lipid extract (see the “Methods” section), 2 μM tOmpA, 10 μM SurA variant (where included) in 20 mM Tris–HCl, 150 mM NaCl, pH 8.0, at 25 °C. Source data are provided as a Source Data file (Supplementary Data 10).

To investigate the role of SurA in mediating BAM function we performed in vitro kinetic assays monitoring the folding of the well-characterised OMP substrate, tOmpA (the β-barrel domain of OmpA (residues 22–192^85^), by BAM. We reconstituted BAM into proteoliposomes formed from *E. coli* polar lipid extract^13,78,86^, and used cold SDS–PAGE assays to measure the rate of BAM-mediated tOmpA folding in the presence or absence of SurA (see the “Methods” section). These experiments showed that in the presence of SurA-WT and BAM, tOmpA can fold rapidly (Table S2) and efficiently into BAM-containing proteoliposomes formed from *E. coli* polar lipid, with yields of folded tOmpA reaching ~100%^86^ (Figs. 4, S11, S12). By contrast, in the absence of SurA-WT, tOmpA is still able to fold into BAM-containing proteoliposomes, but folding is ~6-fold slower, with a reduced final folding yield (~60%) (Figs. 4, S11, and S12). No folding was observed for tOmpA into liposomes lacking BAM in the presence or absence of SurA-WT (‘Empty’) (at least on the timescale of ~18 h) (Figs. 4, S11, and S12). The results highlight the importance of BAM for folding of tOmpA into liposomes formed from *E. coli* polar lipid^13,48,78,86,87^, and demonstrate that in the presence of SurA-WT folding is both more rapid and more efficient than in the absence of the chaperone.

Next, we produced three further SurA variants to investigate the role(s) of its core, P1 and P2 domains in the folding of OMP substrates catalysed by BAM. Variants were created by removing each PPIase domain individually (creating SurA-ΔP1 and SurA-ΔP2), or together (SurA-core) (Fig. 1e). MST binding experiments showed that tOmpA has similar (low µM) affinity for each of the SurA variants, consistent with previous results^83^ (Fig. S13, Table S3), and all of the SurA variants were able to suppress the aggregation of tOmpA under the experimental conditions used in kinetic experiments (Fig. S14). All SurA variants also bind BAM (measured by MST using BAM-containing proteoliposomes) with similar affinity to each other (*K*_d_ ranging from 0.6 to 1.2 μM) (Fig. S15, Table S4) and with that measured for the SurA–WT–BAM interaction in DDM micelles (*K*_d_: 2.6 ± 0.2 µM) (Fig. 1f). These results confirm that BAM binds directly to SurA in vitro, and independently of the presence of the SurA P1 and P2 domains, rather than indirectly via SurA-bound OMP substrates (although an increased affinity in the presence of OMP cannot be ruled out). That deletion of one or both PPIase domains does not substantially alter binding affinity (Fig. S15) accords with the HDX-MS data above which suggest that the core domain contains sites primarily responsible for BAM-binding, and is consistent with data suggesting that the core domain can, for the most part, functionally replace full-length SurA in vivo^27^.

Next, the ability of each SurA variant to facilitate BAM-catalysed folding of tOmpA was measured using kinetic folding assays monitored by cold SDS–PAGE. The results (Fig. 4) showed that deletion of the SurA P1 domain had little effect on the rate of tOmpA folding. In marked contrast, deletion of either P2 (SurA-∆P2), or both PPIase domains (SurA-core), slows the folding kinetics of tOmpA ~3-fold (Table S2, Fig. 4).

Taken together, the kinetic and binding data reveal the striking finding that whereas deletion of the P2 domain from SurA decreases the ability of the chaperone to mediate SurA-BAM-catalysed tOmpA folding, deletion of P1 has little effect, suggesting that each domain plays a different mechanistic role(s) in the delivery of OMPs to BAM for folding. The data also highlight the importance of the SurA core domain for binding both OMPs and BAM, and in promoting OMP folding. Finally and importantly, the data suggest a model in which the SurA P2 domain, which recent data suggests makes few contacts with bound OMP substrates^30,31^, plays an important role in promoting OMP folding via BAM.

### Structural model of the BAM–SurA complex

To obtain a structural model of the BAM–SurA assembly which could explain our experimental data we took advantage of the recently released deep learning protein structure prediction model AlphaFold-Multimer^73,75^. Using the mature sequences for the complete BamABCDE complex and SurA-WT as input, five BAM–SurA models were generated that were similar to each other (average root mean squared deviation (RMSD) between backbone atoms of 1.9 ± 0.8 Å), therefore only a single model is described here (Supplementary Data 3).

**Fig. 5:**
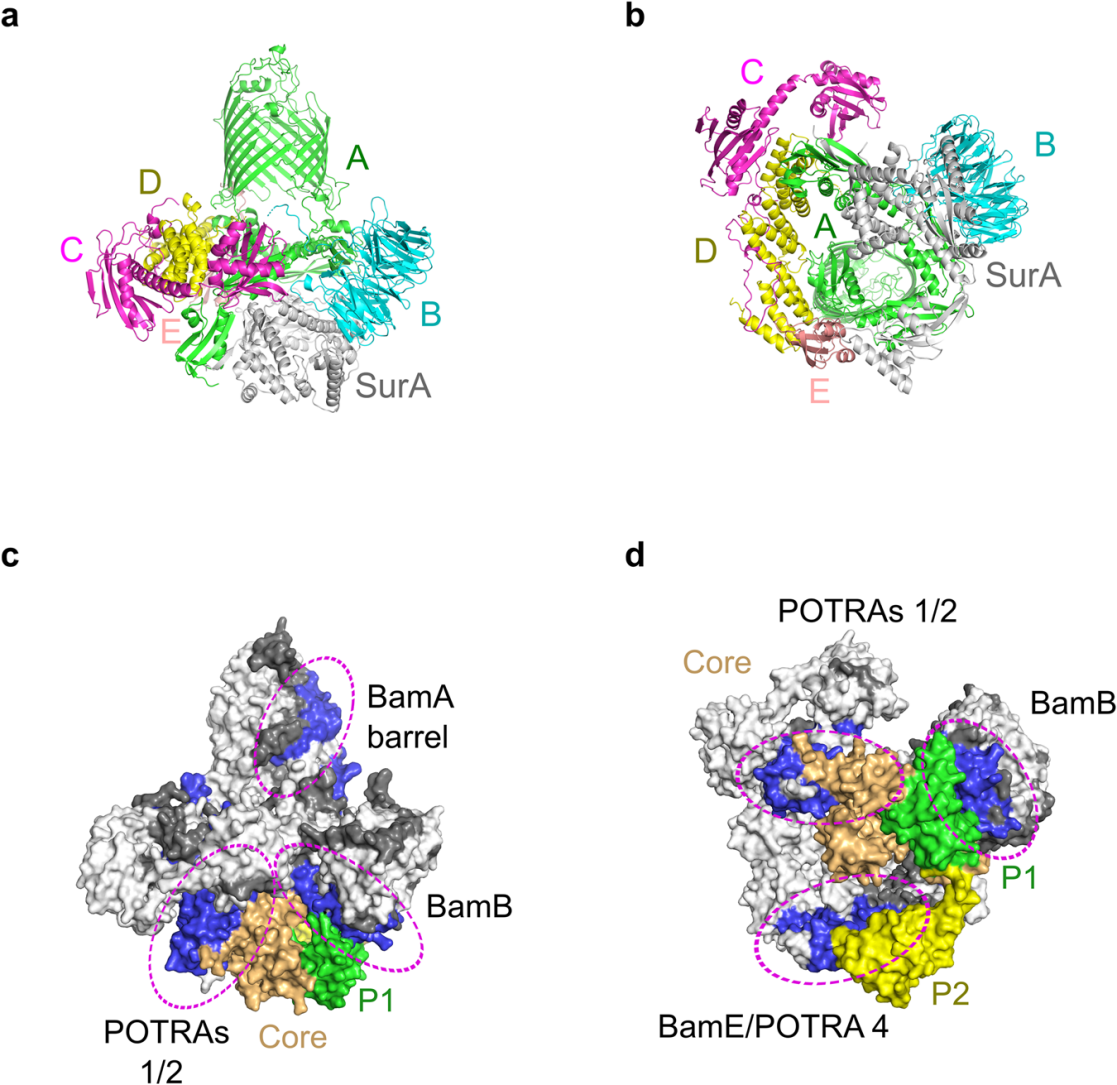
AlphaFold-Multimer generated model of the BAM–SurA complex. **a**, **b** The predicted structure of the BAM–SurA complex coloured by subunit. SurA is in grey. **c**, **d** Surface views of the BAM–SurA complex with regions of HDX protection in the BAM complex upon binding SurA highlighted, and SurA coloured by domain. Regions of BAM that are protected from HDX in the presence of SurA are highlighted in blue. Regions in white show no change in deuterium uptake in the presence of SurA, while those in dark grey denote sequences for which peptides were not detected. Patches of protection from HDX upon SurA binding in the BamA β-barrel domain (adjacent to the lateral gate), POTRAs 1 and 2, BamB, and BamE/POTRA 4 are ringed in magenta. The SurA core, P1 and P2 domains are coloured in orange, green and yellow, respectively. Left panels show a side view of the complex, and right panels a view from the periplasmic face.

The predicted structure of BAM is in the ‘lateral-closed’ state, with a backbone RMSD of 1.9 Å compared with the crystal structure of BAM in a ‘lateral-closed’ state (PDB: 5AYW^12^) (Fig. 5). The per residue Predicted Local Distance Difference Test (pLDDT), a measure of local model confidence^73^, indicates high model confidence in the local structural arrangement for the majority of residues in both BAM and SurA (Fig. S16). Overall, SurA is in an extended conformation without substantial contacts between its domains. By contrast, single molecule FRET, cross-linking and SANS studies showed that the chaperone adopts a broad ensemble of conformers in solution, which involve more intimate contacts between the domains than observed in the predicted state bound to BAM^30,32,33^. Expansion of the chaperone upon binding to BAM is fully consistent with the deprotection observed here by HDX-MS (Figs. 5, S17). In the BAM-SurA AlphaFold model, the SurA core domain interacts extensively with BamA POTRA domains 1 and 2, as well as with BamB, consistent with our HDX results (Figs. 5, S9, S17, Supplementary Video 3, Supplementary Data 4). An intriguing β-augmentation interaction is predicted between β2 of POTRA 1 and five residues in the N-terminal region of SurA which are unstructured or not visible in the SurA crystal structure^28^ (Fig. S18a). The SurA P2 domain is predicted to contact both BamE and BamA POTRA 4, again consistent with our HDX data (Figs. 5 and S18b).

Intriguingly, in the AlphaFold model the SurA P1 domain is released from the core domain compared with the ‘core-P1-closed’ conformation observed in the SurA crystal structure, potentially explaining the deprotection observed at the SurA core–P1 interface observed by HDX-MS (Fig. 3). Instead of contacting the SurA core, P1 contacts BamB on its side facing into the BAM periplasmic ring, wherein HDX protection was again detected. The orientation of the SurA-core domain creates a fascinating, potential direct route for the unfolded OMP to access the BAM periplasmic ring from the SurA OMP-binding cradle^30,31^. Such a model would rationalise the increased efficiency of tOmpA folding via BAM in the presence of SurA, since SurA would facilitate delivery of the OMP to the protective BAM periplasmic ring and orient it for efficient access to the BamA lateral gate.

We examined the predicted alignment error (PAE) between pairs of residues in SurA and each BAM subunit (Fig. S19). This parameter is a measure of the expected error in position between pairs of residues (*x*,*y*) when residue *x* is aligned on residue *y*^75^. An overall low PAE score between pairs of residues in two domains indicates that AlphaFold predicts a well-defined relative position between the domains. Conversely, a high PAE score between pairs of residues in two domains indicates uncertainly in the relative position of the domains^75^. The low PAE scores between SurA core domain residues and residues in BamA POTRA domains 1 and 2, BamB and BamD indicates high confidence in the prediction of a well-defined interaction between the SurA core domain and BAM (Fig. S19a, b, d). Similarly, low PAE scores are observed between residues in the SurA P2 domain and BamE, particularly around the predicted SurA-P2/BamE interface (Fig. S19e). Conversely, AlphaFold predicts no well-defined orientations between the SurA P1 domain and BAM, as high PAE scores are observed between SurA P1 and all BAM subunits (Fig. S19a–e). Such high scores would be consistent with a model for BAM-SurA which involves a dynamic ensemble of conformations for SurA P1 on BAM binding, wherein P1 is released from the core domain (consistent with our HDX-MS data). In such a model ‘core-P1-closed’ states would no longer be populated and P1 may contact different BAM subunits, although no P1-BAM cross-links were found (Fig. S10). Therefore, although in the predicted model SurA P1 contacts BamB, it is possible that the HDX protection in this region of BamB may result from an additional or alternative interaction with the SurA core domain, consistent with the hydrogen exchange observed on BamB in the presence of SurA-core only (Fig. S8).

Mapping our experimental XL-MS data onto the predicted BAM-SurA model we were able to observe satisfied cross-links for only 5 of the 17 observed cross-links between SurA and BAM. These involved the cross-links from SurA to each of the three major regions binding regions on BAM observed by HDX (POTRAs 1–2, BamB and POTRA 4/BamE) (Figs. 2, S20a, b, Table S5, Supplementary Data 6). The presence of many unsatisfied crosslinks (12/17) that are inconsistent with a single structure generated by AlphaFold, suggests the BAM–SurA complex must populate a dynamic conformational ensemble of different structures (Fig. S20c, d), in particular with respect to the location of the SurA P2 domain (Figs. S10, S20). The unsatisfied crosslinks can be separated into three different types (Table S5, Supplementary Data 7): (1) BamC to SurA-core (3 cross-links). These may be explained by dynamic movements of the BamC helix grip domains. High PAE scores are observed between SurA and these domains, which contact BamD and POTRAs 1 and 2 in the predicted model (Fig. 5). The BamC helix grip domains are only fully visible in one BAM structure (BamACDE, PDB: 5D0Q^11^), and in vivo evidence suggests they are surface accessible (at least in a proportion of BAM molecules)^88,89^, therefore the relevance of the predicted location of these domains in the BAM-SurA complex remains to be seen; (2) SurA-core to BamB and BamD (3 cross-links). Cryo-EM data suggests that BamA POTRA domains 1 and 2 are flexible with respect to the other POTRA domains^13,50^, which could facilitate movements of SurA core domain within the BAM periplasmic ring while still bound to POTRAs 1 and 2, potentially bringing the core domain closer to BamB and BamD; and (3) SurA P2 domain (or the linker between P1 and P2 domains) to BamA, BamB and BamD (6 cross-links). Our experimental binding data indicates that the main contribution to affinity for BAM comes from the SurA core domain (Fig. S7). Thus, it is possible that the P2 domain is able to leave (and rebind) to its binding site on BamE/POTRA 4 while SurA remains tethered to BAM via its core domain. The length of the linkers between the domains of SurA are such that the domains can populate a wide range of distances and orientations with respect to one another^30^. Therefore, an unbound SurA P2 domain, while tethered to BAM via the SurA-core domain, could populate a large number of conformations close to the BAM periplasmic ring and possibly explain all of these unsatisfied crosslinks. Taken together, the crosslinking data informs that, while the predicted AlphaFold model is in good agreement with some of our other experimental data, there are additional dynamic movements within the BAM–SurA complex are required to explain all of the HDX and cross-linking data obtained.

Finally, to assess the probability of the BAM–SurA AlphaFold model representing a stable complex in solution we used PDBePISA^90^ to examine the interfaces between SurA and BAM (Tables S6, S7). The BamA-SurA and BamE-SurA interfaces were predicted to be energetically favourable (∆*G*° of −8.3 and −3.6 kcal/mol, respectively), in agreement with our in vitro data indicating that the SurA core and P2 domains are the functionally important domains for BAM-mediated folding of tOmpA (Fig. 4). Conversely, the BamB–SurA interface was predicted to be unfavourable (∆*G*° of 5.9 kcal/mol), consistent with the PAE data predicting no well-defined orientation between P1 and BamB (Fig. S19).

## Discussion

The importance of SurA for cell envelope homoeostasis and virulence, and its role in OMP assembly has long been established^14,16,91^. However, how SurA facilitates OMP biogenesis remained unclear, with possible models including direct transfer of OMPs to BAM from the SurA-bound state, supercomplex formation between BAM, SurA and the Sec holotranslocon, and/or SurA acting merely as a holdase, preventing OMP aggregation in the periplasm which would then permit spontaneous folding of OMPs without requiring SurA-mediated delivery to BAM^5,38,42,67^. A first step in addressing these outstanding questions was to determine whether SurA directly binds BAM and the molecular determinants of SurA’s interaction with BAM. Here, using functional assays, HDX-MS and cross-linking, we provide insights into the molecular details of the interaction between SurA and BAM, demonstrating that there is indeed a direct physical interaction between BAM and SurA, at least in vitro, that is not mediated via OMPs. Using HDX-MS we have mapped interaction sites on both BAM and SurA, showing key roles for the SurA core and P2 domains in contacting the periplasmic region of BAM. Accordingly, using domain deletion variants of SurA in kinetic assays we have revealed a role for both the core and P2 domains in enhancing tOmpA folding via BAM in vitro.

Using AlphaFold-Multimer we generated a model of the SurA–BAM complex (Fig. 5) which is in remarkable agreement with our HDX data, despite being generated independently. Higher resolution data, for example from cryo-EM or X-ray crystallography, will now be needed to define the precise conformation(s) in the complex and how they change during a BAM-mediated functional cycle of OMP folding. Nonetheless, our HDX data clearly indicate the main sites of interaction of the proteins, and show that both BAM and SurA undergo changes in conformations and/or dynamics upon binding. Given the dynamic nature of both BAM and SurA it is likely that multiple conformations are populated in the complex, supported by the fact that our observed crosslinks cannot be satisfied by a single model (Fig. S20, Table S5). Previous in vivo crosslinking and mutagenesis studies have identified that the POTRA 1 domain of BamA, and specifically the region surrounding R64 in the α2 helix^66,92^, is involved in the interaction of BAM with SurA in vivo. Consistent with this, the α2 helix of POTRA 1 is one of the regions of BamA that shows protection in the presence of SurA in our HDX-MS data, and forms a salt bridge with D41 in the SurA core domain in the AlphaFold predicted BAM–SurA model (Table S7).

**Fig. 6:**
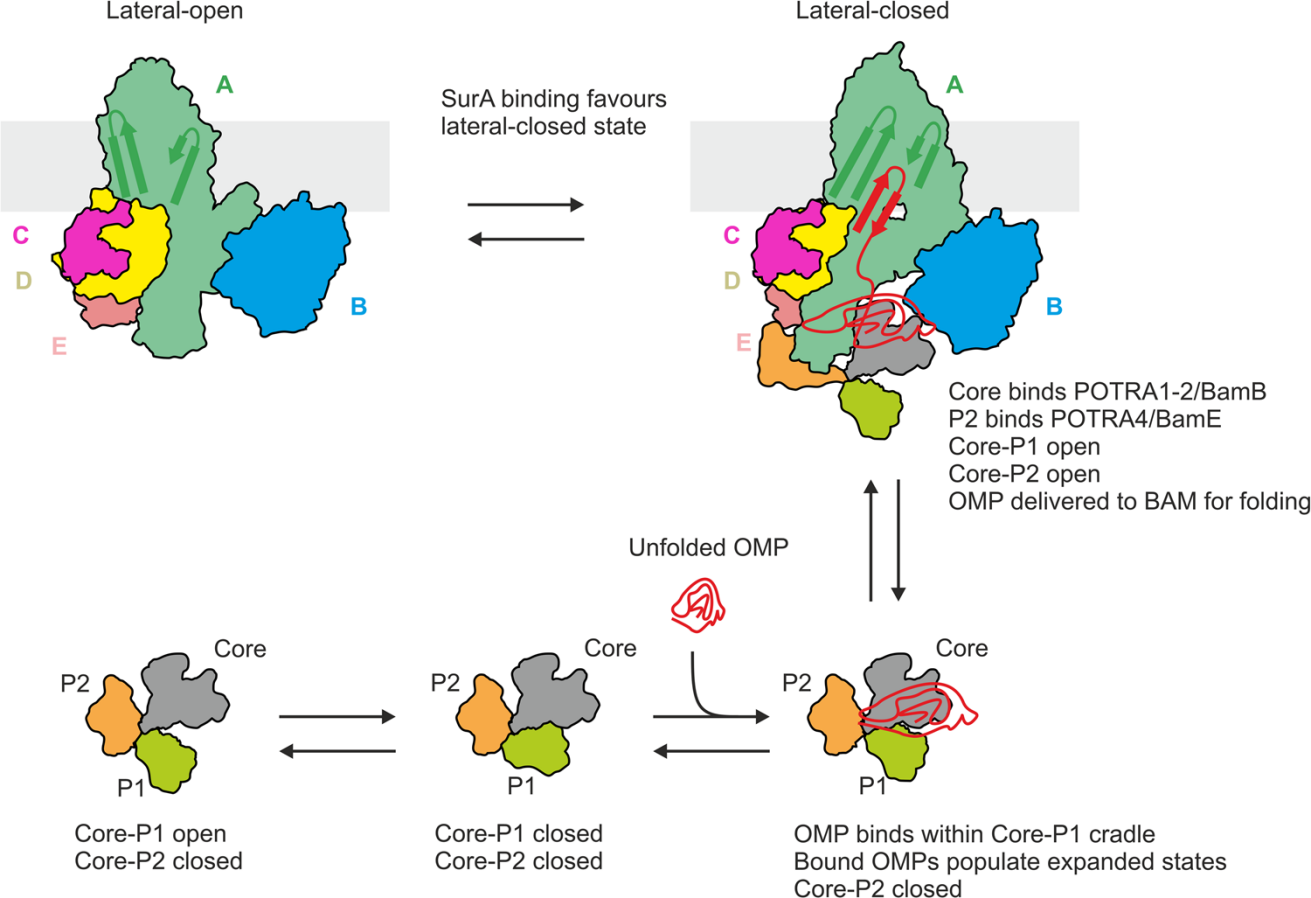
Proposed model of SurA-mediated delivery of OMPs to BAM for folding. In the membrane, BAM is in dynamic equilibrium, populating a number of conformations including those in which the BamA barrel is in either a ‘lateral-open’ or ‘lateral-closed’ conformation (top). SurA is also dynamic, populating ‘Core-P1 open’ and ‘Core-P1 closed’ ensembles of states (bottom). The P2 domain is shown residing close to the core domain in solution (‘Core-P2 closed’). OMPs bind in a cradle formed by the Core and P1 domains, with binding sites primarily located on the core domain^30,31^. OMP binding to SurA causes the OMP to populate expanded states^31,34,35^ The OMP–SurA complex binds to BAM, to form an OMP–SurA–BAM ternary complex, resulting in conformational changes in both SurA (opening of the P1 and P2 domains) and BAM (favouring the lateral-closed state, which we propose to be the OMP acceptor state). The OMP binding cradle of SurA is oriented into the BAM periplasmic ring, allowing release of the unfolded OMP into a protective ‘chaperonin-like’ environment^5^, and presentation of the OMP to the BamA β-barrel for folding in a C- to N-terminus direction via β-strand elongation from β1 of the BamA barrel^5,52,53,59,61,114^. Here, a single β-hairpin of the substrate is depicted bound to β1 of the BamA barrel, however more extensive β-sheet structure in the substrate may begin to form in the periplasm (or at the water–membrane interface) prior to membrane integration^5,53,114,115^, in a manner analogous to a seeded amyloid aggregation reaction^5,116,117^.

Using HDX-MS we identified two regions in the SurA core domain that are protected from HDX in the presence of BAM (Fig. 3). Interestingly, these regions do not overlap with the binding sites for OMP substrates that have been identified previously using XL-MS and HDX-MS^30,31^. This suggests that SurA–OMP binding does not preclude SurA–BAM binding and hence ternary BAM–OMP–SurA complex formation. In addition, we identified multiple regions in SurA that are deprotected in the presence of BAM, spread across all three SurA domains. Notably, a similar region of deprotection at the core–P1 interface of SurA is also observed in the presence of unfolded OMP substrates^30^. This suggests a reorganisation of the domains of SurA upon binding BAM, consistent with a pliable multi-domain protein that responds to the presence of its interaction partners, specifically in the orientation of the P1 and P2 domains relative to the core^30^. Recently, the conformational dynamics and motions of the P1 and P2 domains of apo-SurA relative to the core have been studied by crosslinking^30^, smFRET^30,33^, SANS^32^ and NMR^33^. While the distances and orientations between domains and the populations of different conformational states have not been resolved, a number of features of the conformational landscape have been proposed including that: (1) the P1 and core domains populate conformations in which they are either in close proximity, similar to the crystal structure^28^ (Fig. 1d), or dissociated from one another to populate core-P1 open state(s); and (2) the P2 domain populates conformations in which it resides close to the core domain (unlike the orientation observed in the crystal structure^28^ (Fig. 1d)), and may compete with P1 for binding to the core domain^32^, and/or bind the core domain at multiple sites^33^. In order for SurA to bind to BAM in the manner we propose here, mediated by interactions between P2 and BamE/BamA POTRA 4, ‘P2-open’ states must be populated, and it is tempting to speculate that an ‘unfurling’ of the SurA structural ensemble upon binding to BAM may promote the release of bound clients into the periplasmic ring of BAM so that they can be recognised by the folding machinery and inserted into the OM (Fig. 6).

Our HDX-MS data indicate that SurA binding to the BAM periplasmic ring, and in particular the interaction between the SurA P2 domain and BamE/BamA POTRA 4, triggers structural alterations distal to the binding sites in the lateral gate region of the BamA β-barrel (Figs. 2 and S9). Structures of BAM determined to date suggest that BAM populates at least two main states, in which ‘lateral-open’ or ‘lateral-closed’ states of the BamA barrel at the β1–β16 seam appear to be coordinated with a rigid body rotation of the POTRA domains^10–13^ (Fig. 1b, c). Importantly, access to the periplasmic face of the BamA β-barrel is occluded by POTRA 5 when the barrel seam is in the lateral-open conformation, suggesting a role for conformational cycling in the functional mechanism of BAM (Fig. 1b, c). In support of this, constraint of BamA barrel conformational dynamics by antibody binding to extracellular loop 4, or by disulfide tethering of β1–β16, extracellular loop 1 to extracellular loop 6, or POTRA 5 to intracellular turn 4, are lethal in vivo^11,51^, and slow BAM-mediated OMP folding kinetics in vitro^13,86^. This suggests a role for structural plasticity in both the BamA barrel and POTRA domains in BAM function. Restricting the conformational freedom in the hinge region between POTRA domains 2 and 3 also results in impaired BAM function in vivo^93^. It was previously proposed that POTRA 2-POTRA 3 hinging motions may serve to mediate access to the BAM periplasmic ring, to accommodate nascent OMPs of a range of sizes, and/or to regulate interactions with BamB and BamD^93^. Our HDX-MS data suggest hinging motions between POTRAs 2 and 3 may also be involved in regulating allosteric changes in the BamA β-barrel upon binding of SurA, presumably to prime the BamA barrel to engage OMP substrates for folding and insertion into the OM.

Multiple lines of evidence suggest an allosteric connection between the periplasmic region of BAM and the BamA β-barrel domain including: (1) the correlation between lateral opening of the BamA β-barrel gate and a rotational conformational change in the BAM periplasmic ring in BAM structures^10–13^; (2) a mutation in BamD (R197L) or deletion of BamE affects the surface exposure of BamA extracellular loop 6 (eL6)^94,95^; (3) mutations in BamA eL6 suppress OMP assembly defects caused by the periplasmic BamA^E373K^ mutation^96^; (4) substitutions in the BamA β-barrel domain rescue the synthetic lethal phenotype of a *∆bamB∆bamE* strain^97^; and (5) mutations in the BamA β-barrel domain can overcome OMP assembly defects in BamA barrel-POTRA chimeras^98^. The combined evidence from structural studies suggests that the lateral-closed conformation of BamA (also known as ‘inward-open’), in which the BamA barrel is accessible from the periplasm, may be the acceptor state for receipt of unfolded OMPs^59–61,63,65^. Here, we have shown that protection from HDX occurs in the BamA barrel in the presence of SurA, suggesting that binding of the chaperone to the periplasmic region of BAM may favour the BamA lateral-closed state. Recent structural and simulation evidence suggest that the lateral-open conformation of BAM is the major populated state in the absence of SurA^62^. Thus, we propose a model in which SurA acts to ready the BAM machinery for OMP folding by modulating the BAM conformational ensemble to favour the acceptor state. Significant levels of protection are not observed in the BamA β-barrel in the presence of SurA-core, despite the proteins interacting with a similar *K*_d_ (Figs. 1f and S7), suggesting that in the absence of the SurA P2 domain allosteric signalling is less efficiently conveyed, consistent with the observation that BAM-mediated folding of tOmpA in the presence of SurA-core is slower than in the presence of SurA-WT (Fig. 4).

As well as the observation that SurA crosslinks to BamA in vivo^14,22,66^, recent data have suggested that SurA can simultaneously interact with BamB and BamA in vivo^77^. The physical interaction between BamB and SurA demonstrated here could provide a rationale for these observations, as well as the observation that BAM complexes lacking BamB are less efficient catalysts of SurA-assisted OMP folding in vitro^36^. Further, our results are consistent with genetic results linking SurA and BamB: the cellular effects of SurA or BamB deletion are similar, and loss of both SurA and BamB leads to a severe synthetic phenotype^9^. Given that BamB has been implicated in the formation of self-associated ‘BAM islands’^22,89,99,100^, the severity of the ∆*bamB* phenotype may also be influenced by a reduction in these BAM assemblies in the OM, as well as the loss of functional interaction with SurA.

Previous evidence has implicated BamE in modulating BamA structure^94,95,101^. Genetic deletion of BamE increases the susceptibility of BamA to degradation by proteinase K^94^, and leads to increased surface exposure of eL6^95^. Recent data from neutron reflectrometry experiments also show conformational changes in BamA in the presence of BamE^101^, and computational analyses have revealed co-evolution between residues in BamE and residues in SurA, including those in the P2 domain, suggesting that this interaction is evolutionarily conserved^102^. Consistent with these observations we show here a direct interaction between SurA and BamE using both HDX-MS and XL-MS, providing direct support both for the structural model of SurA–BAM herein reported (Fig. 4), as well as the functional importance of the interaction within the complex.

Together, our results enable a model to be presented of how SurA binds its clients, and delivers them to BAM for folding and insertion into the OM. SurA is known to bind and prevent OMPs from aggregation, and also to modulate the conformational landscape of unfolded OMPs to aid folding^103^, most likely via the creation of extended states in their clients^5,31,34,35^. Here we propose that SurA, as part of the ‘donor’–substrate complex (SurA–OMP), binds directly to BAM, shifts the equilibrium position of BAM towards the lateral-closed (‘inward-open’) acceptor state, to allow the incoming OMP access to the active BamA catalyst (Fig. 6). Alongside, binding of SurA to BAM leads to unfurling of the SurA PPIase domains from the core domain, with the P2 domain reinforcing binding to BAM (via avidity), and potentially priming BAM to commence catalysis, coincident with the commencement of client release from SurA. How the OMP is translocated to the BamA β1 strand to initiate folding^5,53,59,61^ remains unclear, but this could involve sequential binding to different POTRA domains via β-augmentation^104,105^, initial binding to BamD^106,107^, or direct delivery of the β-signal to its unique binding pocket involving β1 of BamA and the membrane^61,65^. Such interactions in a transport chain, in which ‘donor’ and ‘acceptor’ proteins each modulate the conformations of the other, may be a general mechanistic feature of the transfer of unfolded/partially folded proteins between chaperones and other protein machineries in the periplasm, especially since this compartment lacks ATP that is used to choreograph chaperone-mediated folding events in other cellular compartments^108^. Such a mechanism also rationalises how efficient protein folding can be brought about in the periplasm wherein weak binding of OMPs to SurA, and weak binding of SurA to BAM, can nonetheless result in efficient chaperoning, delivery, folding and insertion of OMPs into the OM without the need for an external energy source.

## Methods

### Plasmids

The plasmid for tOmpA encoding the mature sequence of the β-barrel domain of OmpA (residues 22–192) in pET11a was kindly provided by Karen Fleming (John Hopkins University, USA)^87^. To create the Cys-tOmpA plasmid, a Cys residue was added immediately following the N-terminal Met residue using mutagenesis. Expression plasmids pET28b containing the mature SurA sequence (residues 21–428) preceded by an N-terminal 6x His-tag and thrombin-cleavage site (pSK257)^36^ and plasmid pJH114 containing BamABCDE with a C-terminal 8x His-tag on BamE^78^, were kind gifts from Daniel Kahne (Harvard University, USA) and Harris Bernstein (NIH, USA), respectively. The thrombin-cleavage site was changed to a TEV-cleavage site using mutagenesis, yielding SurA with the N-terminal sequence MGSS(H)_6_SSGENLYFQG. SurA domain deletion variants SurA-∆P1, SurA-∆P2, and SurA-core were constructed by removing residues 172–280, 281–389 and 172–389, respectively, as described previously^83^. For measurements of binding to BAM using MST, a cysteine residue was added immediately following the TEV cleavage site to create the Cys-SurA-WT, Cys-SurA-∆P1, Cys-SurA-∆P2, and Cys-SurA-core plasmids. All mutagenesis was performed using Q5 site-directed mutagenesis (NEB). A vector (pMHTDelta238) containing His-tagged TEV protease fused with MBP which is removed in vivo by autocleavage, was obtained from DNASU (Clone TvCD00084286).

### Expression and purification of SurA and SurA variants

Plasmids containing wild-type SurA or SurA variant genes were transformed into BL21(DE3) cells (Stratagene). Cells were grown in LB medium supplemented with 30 µg/mL kanamycin at 37 °C with shaking (200 rpm) until an OD_600_ of ~0.6 was reached. The temperature was subsequently lowered to 20 °C, and expression induced with 0.4 mM IPTG. After ~18 h, cells were harvested by centrifugation, resuspended in 25 mM Tris–HCl, pH 7.2, 150 mM NaCl, 20 mM imidazole, containing EDTA-free protease inhibitor tablets (Roche), and lysed using a cell disrupter (Constant Cell Disruption Systems). The cell debris was removed by centrifugation (20 min, 4 °C, 39,000×*g*), and the lysate was applied to 5 mL HisTrap columns (GE Healthcare). The columns were washed with 25 mM Tris–HCl, pH 7.2, 150 mM NaCl and 20 mM imidazole, followed by 25 mM Tris–HCl, 6 M Gdn–HCl, pH 7.2 (to denature the SurA on-column). After washing with 25 mM Tris–HCl, 150 mM NaCl, pH 7.2, SurA was eluted with 25 mM Tris–HCl, 150 mM NaCl, 500 mM imidazole, pH 7.2. The eluate was dialysed against 25 mM Tris–HCl, 150 mM NaCl, pH 8.0 overnight, and the following day TEV protease^49^ (ca. 0.5 mg) and 0.1% (v/v) β-mercaptoethanol were added. The cleavage reaction was left to proceed overnight at 4 °C on a tube roller. The cleavage reaction was again applied to the 5 mL HisTrap columns (GE Healthcare) to remove the cleaved His-tag and His-tagged TEV protease. The unbound, cleaved SurA product was dialysed extensively against 25 mM Tris–HCl, 150 mM NaCl, pH 8.0, before being concentrated to ~200 µM with Vivaspin 20 concentrators (Sartorius; 5 kDa MWCO), aliquoted, snap-frozen in liquid nitrogen and stored at −80 °C. Protein concentrations were determined spectrophotometrically. N-terminal Cys-containing variants (Cys-SurA-WT, Cys-SurA-∆P1, Cys-SurA-∆P2, and Cys-SurA-core) were purified as detailed above, except for the addition of 1 mM DTT to all buffers in the purification procedure, up until the elution step.

### Expression and purification of the BAM complex

BamABCDE was expressed in *E. coli* BL21(DE3) cells from plasmid pJH114^78^ (kindly provided by Harris Bernstein) and was purified using a combination of Ni-affinity and size-exclusion chromatography as previously described^13^.

### Expression and purification of TEV protease

The vector pMHTDelta238 containing His-tagged TEV fused with MBP which is removed in vivo, by autocleavage, transformed into BL21-CodonPlus[DE3]-RIPL cells (Stratagene, UK). Cells were grown in LB medium containing 50 µg/mL kanamycin at 37 °C with shaking (200 rpm) until the culture reached an OD_600_ of ~0.6. The temperature was then lowered to 30 °C and protein expression induced with 0.5 mM IPTG. After ~4 h the cells were harvested by centrifugation, resuspended in 25 mM sodium phosphate buffer, pH 8.0, 200 mM NaCl, 10% (v/v) glycerol, 25 mM imidazole, 1 mM phenylmethylsulfonyl fluoride (PMSF), 2 mM benzamidine, ~0.02 mg/mL DNase (Sigma, UK), and lysed by sonication (6 × 30 s bursts with 1 min cooling on ice between each sonication). The lysate was centrifuged to remove cell debris (20 min, 4 °C, 39,000 × *g*), applied to Ni^2+^ Sepharose beads (GE Healthcare) and washed twice with 25 mM sodium phosphate buffer, pH 8.0, 200 mM NaCl, 10% (v/v) glycerol, 25 mM imidazole. His-tagged TEV was eluted with 25 mM sodium phosphate buffer, pH 8.0, 200 mM NaCl, 10% (v/v) glycerol, 500 mM imidazole. The eluate was filtered (0.2 µM syringe filter, Sartorius, UK) and gel filtered on a HiLoad Superdex 75 26/60 column (GE Healthcare) equilibrated with 25 mM sodium phosphate buffer, pH 8.0, 200 mM NaCl, 25 mM imidazole, 10% (v/v) glycerol, 5 mM β-mercaptoethanol. Peak fractions were concentrated to ~1 mg/mL using Vivaspin 20 (5 kDa MWCO) concentrators (Sartorius), aliquoted, snap-frozen in liquid nitrogen and stored at −80 °C.

### Expression and purification of tOmpA and Cys-tOmpA

OMPs were purified using a method adapted from ref. ^109^. Briefly, *E. coli* BL21[DE3] cells (Stratagene) were transformed with a pET11a plasmid containing the gene sequence of the mature OMP. Overnight cultures were subcultured and grown in LB medium (500 mL) supplemented with carbenicillin (100 μg/mL), at 37 °C with shaking (200 rpm). Protein expression was induced with IPTG (1 mM) once an OD_600_ of 0.6 was reached. After 4 h the cells were harvested by centrifugation (5000 × *g*, 15 min, 4 °C). The cell pellet was resuspended in 50 mM Tris–HCl pH 8.0, 5 mM EDTA, 1 mM PMSF, 2 mM benzamidine, and the cells were subsequently lysed by sonication. The lysate was centrifuged (25,000 × *g*, 30 min, 4 °C) and the insoluble material was resuspended in 50 mM Tris–HCl pH 8.0, 2% (v/v) Triton X-100, before being incubated for 1 h at room temperature, with gentle agitation. The insoluble material was pelleted (25,000 × *g*, 30 min, 4 °C) and the inclusion bodies washed twice by resuspending in 50 mM Tris–HCl pH 8.0 followed by incubation for 1 h at room temperature with gentle agitation, and then collected by centrifugation (25,000 × *g*, 30 min, 4 °C). The inclusion bodies were solubilised in 25 mM Tris–HCl, 6 M Gdn–HCl, pH 8.0 and centrifuged (20,000 × *g*, 20 min, 4 °C). The supernatant was filtered (0.2 µM syringe filter, Sartorius) and the protein was purified using a Superdex 75 HiLoad 26/60 gel filtration column (GE Healthcare) equilibrated with 25 mM Tris–HCl, 6 M Gdn–HCl, pH 8.0. Peak fractions were concentrated to ∼500 µM using Vivaspin 20 (5 kDa MWCO) concentrators (Sartorius), and the protein solution was snap-frozen in liquid nitrogen and stored at −80 °C.

### Hydrogen–deuterium exchange mass spectrometry (HDX-MS)

HDX experiments were conducted using an automated robot (LEAP Technologies) that was coupled to an Acquity M-Class LC with HDX manager (Waters). Samples contained 0.02% n-Dodecyl-β-d-Maltoside (DDM), in 10 mM potassium phosphate, pH 8.0. Experiments examining HDX protection in BAM contained 8 µM BAM and 10 µM SurA-WT or SurA-core. Experiments examining HDX protection in SurA-WT in the presence of BAM contained contained 8 µM SurA-WT and 10 µM BAM. The automated robot was used to transfer 95 μL of deuterated buffer (10 mM potassium phosphate, pD 8.0, 0.02 % DDM) to 5 μL of protein-containing solution, and the mixture was subsequently incubated at 4 °C for 0.5, 2, 30, 120 min. Three replicate measurements were performed for each time point and condition studied. 100 μL of quench buffer (10 mM potassium phosphate, 0.05% DDM, pH 2.2) was added to 50 μL of the labelling reaction to quench the reaction. 50 μL of the quenched sample was injected onto immobilised pepsin and aspergillopepsin columns (Affipro) connected in series (20 °C). A VanGuard Pre-column [Acquity UPLC BEH C18 (1.7 μm, 2.1 mm × 5 mm, Waters)] was used to trap the resultant peptides for 3 min. A C18 column (75 μm × 150 mm, Waters, UK) was used to separate the peptides, employing a gradient elution of 0–40% (v/v) acetonitrile (0.1% v/v formic acid) in H_2_O (0.3% v/v formic acid) over 7 min at 40 μL min^−1^. The eluate from the column was infused into a Synapt G2Si mass spectrometer (Waters) that was operated in HDMS^E^ mode. The peptides were separated by ion mobility prior to CID fragmentation in the transfer cell, to enable peptide identification. Deuterium uptake was quantified at the peptide level. Data analysis was performed using PLGS (v3.0.2) and DynamX (v3.0.0) (Waters). Search parameters in PLGS were: peptide and fragment tolerances = automatic, min fragment ion matches = 1, digest reagent = non-specific, false discovery rate = 4. Restrictions for peptides in DynamX were: minimum intensity = 1000, minimum products per amino acid = 0.3, max. sequence length = 25, max. ppm error = 5, file threshold = 3. Peptides with statistically significant increases/decreases in deuterium uptake were identified using the software Deuteros^110^. Deuteros was also used to prepare Woods plots. The raw HDX-MS data have been deposited to the ProteomeXchange Consortium via the PRIDE/partner repository with the dataset identifier PXD030268. A summary of the HDX-MS data, as per recommended guidelines^111^, is shown in Table S8.

### Chemical crosslinking mass spectrometry (XL-MS)

10 μM of BAM with 10 μM of SurA was buffer exchanged into 10 mM potassium phosphate, 150 mM sodium chloride, pH 8.0, 0.02% DDM. DSBU was added at a final concentration of 500 μM and the crosslinking reaction was allowed to proceed for 30 min at room temperature. The reaction was quenched by adding 20 mM Tris (pH 7.5). The crosslinked proteins were precipitated using chloroform–methanol, and the proteins were resuspended in rapigest (Waters) (1 % (w/v) in 50 mM ammonium bicarbonate pH 8, 10 μL). Dithiothreitol (50 mM in 50 mM ammonium bicarbonate pH 8, 10 μL) was then added and the mixture was incubated at 37 °C for 1 h. Next, iodoacetamide (100 mM in 50 mM ammonium bicarbonate pH 8, 10 μL) was added and the mixture was incubated at 37 °C for a further 1 h. Finally, trypsin was added to the reduced and alkylated protein (1:50 w/w enzyme:protein, Promega) along with 50 mM ammonium bicarbonate pH 8 (70 μL) and the mixture was then incubated overnight at 37 °C. The peptides were desalted using Sep-Pak tC18 cartridges (Waters), according to the manufacturer’s instructions. The desalted peptides were evaporated to dryness, and resuspended in 0.1% trifluoroethanol (50 μL). The peptides were then analysed by liquid chromatography–mass spectrometry (LC–MS) on an Orbitrap Exploris 240 mass spectrometer (Thermo Fisher).

Peptides (3 μL) were injected onto a 30 cm capillary emitter column (inner diameter 75 μm, packed with 3-μm Reprosil-Pur 120 C18 media, Dr. Maisch) prepared in-house, and separated by gradient elution of 2–30% (v/v) solvent B (0.1% (v/v) formic acid in acetonitrile) in solvent A (0.1% (v/v) formic acid in water) over 60 min at 300 nL min^−1^. The mass spectrometer was operated in data-dependent acquisition mode with precursor fragmentation performed by higher-energy C-trap dissociation. Each high-resolution scan (*m/z* range 500–1500, *R*  =  60,000) was followed by 10 product ion scans (*R*  =  15,000), using stepped normalised collision energies of 27%, 30% and 33%. Only precursor ions with charge states 3–7+ (inclusive) were selected for tandem MS. The dynamic exclusion was set to 60 s. Cross-link identification was performed using MeroX v2.0.1.4. A maximum of two of the four marker ions, corresponding to fragmentation of the crosslinker, were allowed to be missing in the database search, the mass tolerances were set to 10 ppm and the false discovery rate was set to 1%. Manual verification of all spectra was performed to ensure correct assignment and that a significant degree of sequence coverage of each crosslinked peptide was present in the spectrum. Raw XL-MS data have been deposited to the ProteomeXchange Consortium the PRIDE partner repository with the dataset identifier PXD030209. A reporting summary (based on community guidelines^112^) can be found (Supplementary Data 5).

### Reconstitution of the BAM complex into proteoliposomes

*E. coli* polar lipid extract, purchased as powder from Avanti Polar Lipids (Alabaster, AL), was dissolved in 80:20 (v/v) chloroform/methanol at 20 mg/mL. Appropriate volumes were dried to thin films in clean Pyrex tubes at 42 °C under N_2_ gas, and were further dried by vacuum desiccation for at least 3 h. The BAM complex in TBS pH 8.0, 0.05% (w/v) DDM was mixed with *E. coli* polar lipid extract films solubilised in TBS pH 8.0, 0.05% (w/v) DDM in a 1:2 (w/w) ratio. Empty liposomes were prepared by mixing lipid with an equivalent volume of buffer. To remove detergent and promote liposome formation, the mixtures were dialysed against 2 L of 20 mM Tris–HCl pH 8.0, 150 mM KCl using 12–14 kDa MWCO D-Tube™ Maxi Dialyzers (Merck) at room temperature for 48 h with a total of four buffer changes. Following dialysis, the proteoliposomes were pelleted twice by ultracentrifugation at 100,000 × *g* for 30 min at 4 °C and were resuspended in TBS pH 8.0. Protein concentration was determined using a BCA assay (Thermo Scientific) and successful reconstitution was confirmed by SDS–PAGE.

### BAM-mediated folding of OMPs by SDS–PAGE band-shift assays

Solutions of 20 µM tOmpA denatured in 20 mM Tris–HCl, 150 NaCl, pH 8.0 (TBS) containing 8 M urea were diluted 5-fold into a 20 µM solution of SurA. This mixture was then immediately diluted 2-fold into BAM-containing or empty liposomes to initiate the folding reaction, maintained at 25 °C. Final concentrations were 1 µM BAM, 2 µM tOmpA, 10 µM SurA, 0.8 M urea in TBS pH 8.0. Samples of the folding reaction were taken periodically and were quenched in SDS–PAGE loading buffer (final concentrations: 50 mM Tris–HCl pH 6.8, 10% (v/v) glycerol, 1.5% (w/v) SDS, 0.001% (w/v) bromophenol blue). The samples, including a boiled control (5 min at >95 °C), were run on 15% (w/v) Tris–tricine gels. The gels were stained using InstantBlue™ (Experion) and imaged using an Alliance Q9 Advanced gel doc (UVITEC, Cambridge, UK). Folded and unfolded band intensities were quantified using ImageJ and were plotted as a fraction folded (*I*_Folded_/(*I*_Folded_ + *I*_Unfolded_)) against time. Folding data were fitted to a single exponential function in Igor Pro (V7):

$$y=A\cdot {e}^{-{k}_{{\rm {{obs}}}}t}+\,c$$

where *k*_obs_ is the observed rate constant, *A* is its associated amplitude, and *c* is a constant.

### Nephelometry

tOmpA was buffer exchanged into TBS pH 8.0 containing 8 M urea. Aggregation assays were initiated by diluting each protein to a final concentration of 2 μM in 0.8 M urea TBS pH 8.0 in the presence or absence of 10 µM SurA variant (SurA-WT, SurA-∆P1, SurA-∆P2, or SurA-core). A positive control for aggregation containing 2 µM tOmpA, 0.24 M urea in TBS pH 8.0 was included. The total volume in each sample was 50 µL. The samples were measured in 96-well half area plates (Corning Product No. 3881) and light scattering monitored using a Nephelostar (BMG Labtech GmbH) excited at 635 ± 10 nm at 25 °C. Measurements were made for 2 h. The signal of a buffer blank was subtracted, and the minimum value in each data set was set to zero.

### Labelling of Cys-tOmpA with Alexa Fluor 488

Purified Cys-tOmpA was covalently labelled with Alexa Fluor 488 dye via maleimide chemistry. A sample containing 200 μM Cys-tOmpA in 25 mM Tris–HCl, 6 M Gdn–HCl, pH 7.2, was incubated with 10 mM DTT for 30 min. This sample was subsequently buffer exchanged into 25 mM Tris–HCl, 6 M Gdn–HCl, pH 7.2 (that had been sparged for 15 min with nitrogen gas) using Zeba spin desalting columns (Thermo Fisher Scientific). Alexa Fluor 488 C5 maleimide (Thermo Fisher Scientific) (10 mg/mL dissolved in DMSO) was immediately added to the tOmpA sample at a final concentration of 2 mM. The total sample volume was 480 µL. The labelling reaction was kept at 25 °C for 1 h then left overnight at 4 °C. The reaction was then loaded onto a Superdex Peptide 10/300 column (GE Healthcare) equilibrated with 6 M Gdn–HCl, 25 mM Tris–HCl, pH 7.2 to remove the excess free dye. Samples were collected every 1 mL and peak protein fractions tested for dye labelling using a Nanodrop 2000 (Thermo Fisher Scientific). Samples containing labelled tOmpA were snap-frozen using liquid nitrogen and stored at −80 °C until required.

### Labelling of Cys-SurA-WT, Cys-SurA-∆P1, Cys-SurA-∆P2, and Cys-SurA-core with Alexa Fluor 488

For each SurA variant, the protein was diluted to a concentration of 50 μM in 25 mM Tris–HCl, pH 7.2, 150 mM NaCl, 5 mM DTT. The protein solution was incubated for 30 min at room temperature before being buffer exchanged into 25 mM Tris–HCl, pH 7.2, 150 mM NaCl, 1 mM EDTA using 7 kDa MWCO Zeba spin desalting columns (Thermo Fisher Scientific). A ten-fold molar excess of Alexa Fluor 488 C5 maleimide (Thermo Fisher Scientific) was then added and the samples incubated for 2 h at room temperature with gentle rocking. The reaction was quenched with a 10-fold molar excess (over Alexa Fluor 488 C5) of β-mercaptoethanol. Protein was separated from unbound dye by size exclusion chromatography on a Superdex 200 10/300 GL column (GE Healthcare, UK) equilibrated with 50 mM Tris–HCl, 150 mM NaCl, pH 8.0. Fractions containing labelled protein were combined, snap-frozen in liquid nitrogen and stored at −80 °C.

### Microscale thermophoresis analysis of tOmpA binding to SurA domain variants

Alexa Fluor 488-labelled tOmpA was buffer exchanged into 8 M urea and 20 mM Tris–HCl, pH 8.0. A stock of 200 μM SurA-WT, SurA-∆P1, SurA-∆P2 or SurA-core in 20 mM Tris–HCl, pH 8.0 was used to create a serial dilution (100 μM–3 nM). A 1 µM stock of Alexa Fluor 488-labelled tOmpA was first diluted to 200 nM with 20 mM Tris–HCl, pH 8.0, then added to SurA variant samples to give final concentrations of 100 nM tOmpA and 0.8 M urea in 20 mM Tris–HCl, pH 8.0. The samples were loaded into premium-coated capillaries (NanoTemper Technologies GmbH, Germany) and measured using Monolith NT.115 (NanoTemper Tech.) at 25 °C. Data were fitted to a Hill Eq. (1):

1 $${S}_{{{ {obs}}}}=\,{S}_{{ {U}}}+\left(\,\frac{\left({S}_{{ {B}}}-\,{S}_{U}\right)}{1+\,{\left(\frac{{K}_{ {{d,{app}}}}}{\left[L\right]}\right)}^{n}}\right)$$

where *S*_*obs*_ is the observed signal; *S*_*U*_ and *S*_*B*_ are the signal of the unbound and bound state, respectively; *K*_*d*_,_*app*_ is the apparent *K*_*d*_; *L* is the ligand, which in these experiments is the SurA variant; and *n* is the Hill coefficient. Fits and plots were made with in-house Python 3 scripts and made use of the SciPy and Matplotlib libraries.

### Microscale thermophoresis analysis of SurA domain variants binding to BAM

#### BAM in DDM

A serial dilution of the BAM complex in 0.05% (w/v) DDM, TBS, pH 8.0 (51 μM–1.6 nM) was prepared. Alexa Fluor 488-labelled Cys-SurA-WT or Cys-SurA-core in TBS, pH 8.0 was added to a final concentration of 400 nM in all samples.

#### BAM in proteoliposomes

A serial dilution of BAM reconstituted in proteoliposomes composed of *E. coli* polar lipid extract (25 µM–1 nM) was prepared. Alexa Fluor 488-labelled Cys-SurA-WT, Cys-SurA-∆P1, SurA-∆P2 or SurA-core in TBS, pH 8.0 was added to a final concentration of 400 nM in all samples.

The samples were loaded into premium-coated capillaries (NanoTemper Technologies GmbH, Germany) and measured using Monolith NT.115 (NanoTemper Tech.) at 25 °C. Data were fitted to a 1:1 quadratic binding Eq. (2):

2 $${S}_{{ {{obs}}}}=\,{S}_{{ {U}}}+({S}_{{ {B}}}-\,{S}_{{ {U}}})\times \,\frac{\left(\left[R\right]+\left[L\right]+\,{K}_{{ {d}}}\right)\,\pm \sqrt{{(\left[R\right]+\left[L\right]+{K}_{{ {d}}})}^{2}-4[R][L]}}{2[R]}$$

where *S*_*obs*_ is the observed signal; *S*_*U*_ and *S*_*B*_ are the signal of the unbound and bound state, respectively; *R* is the receptor, which in these experiments is the SurA variant; *L* is the ligand, which in these experiments is the BAM complex; and *K*_*d*_,_*app*_ is the dissociation constant. Fits and plots were made with in-house Python 3 scripts and made use of the SciPy and Matplotlib libraries.

### Generation of models of the BAM–SurA complex

To produce predictions of the structure of the BAM–SurA complex we used Alphafold-Multimer (v2.1.0) installed on a local workstation, using the reduced databases as described at https://github.com/deepmind/alphafold. The root mean squared deviation (RMSD) between the five models generated were calculated by taking the mean and standard deviation of the backbone RMSDs between all pairwise combinations of models. Colouring of the complex by pLDDT score was performed using a Python 3 script for PyMOL ‘colour_af_plddts.py’ available at https://github.com/BobSchiffrin/pymol_scripts. Inter-protein DSBU crosslinks were visualised on the BAM–SurA structural model (and their Cα–Cα distances calculated) using the PyXlinkViewer plugin for PyMOL^113^ available at https://github.com/BobSchiffrin/PyXlinkViewer.

### Reporting summary

Further information on research design is available in the Nature Research Reporting Summary linked to this article.

## Supplementary information

Supplementary Information

Description of additional Supplementary Files

Supp Video 1

Supp Video 2

Supp Video 3

Supplementary Data 1

Supplementary Data 2

Supplementary Data 3

Supplementary Data 4

Supplementary Data 5

Supplementary Data 6

Supplementary Data 7

Supplementary Data 8

Supplementary Data 9

Supplementary Data 10

Supplementary Data 11

Reporting Summary

## Data Availability

The raw MS data have been deposited to the ProteomeXchange Consortium via the PRIDE/partner repository with the dataset identifiers PXD030268 (HDX-MS) and PXD030209 (XL-MS). Source data for MST experiments shown in Figs. 1, S7, S13, and S15 can be found in Supplementary Data 8. Source data for deuterium uptake plots shown in Figs. 2, 3, and S9 can be found in Supplementary Data 9. Source data for tOmpA folding experiments shown in Figs. 4, S11, and S12 can be found in Supplementary Data 10. Source data for nephelometry experiments shown in Fig. S14 can be found in Supplementary Data 11.
