# Supplementary material for: Dynamic interplay between the periplasmic chaperone SurA and the BAM complex in outer membrane protein folding
Source: Commun Biol. 2022 Jun 8;5:560. doi: 10.1038/s42003-022-03502-w (PMC9177699; doi:10.1038/s42003-022-03502-w)
Supplement: Supplementary file 1 — Supplementary Information [file 42003_2022_3502_MOESM1_ESM.pdf]

Supplementary Information

Dynamic interplay between the periplasmic chaperone SurA and the BAM complex in outer membrane protein folding

Bob Schiffrin, Jonathan M. Machin, Theodoros K. Karamanos, Anastasia Zhuravleva, David J. Brockwell, Sheena E. Radford*, Antonio N. Calabrese*

Astbury Centre for Structural Molecular Biology, School of Molecular and Cellular Biology, University of Leeds, Leeds LS2 9JT, United Kingdom

*Corresponding authors: s.e.radford@leeds.ac.uk and a.calabrese@leeds.ac.uk

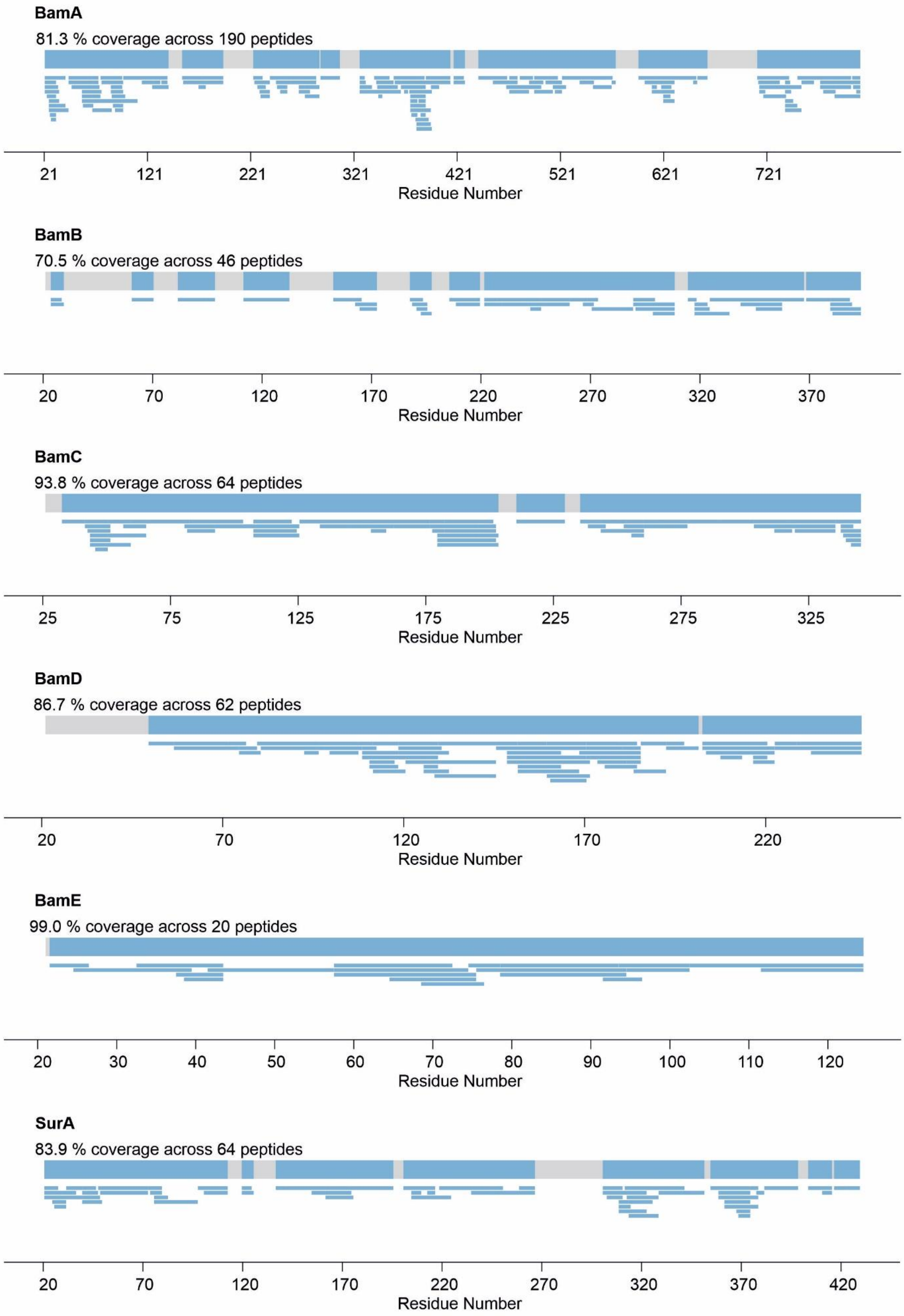
BamA
81.3 % coverage across 190 peptides
21
121
221
321
421
521
621
721
Residue Number
BamB
70.5 % coverage across 46 peptides
20
70
120
170
220
270
320
370
Residue Number
BamC
93.8 % coverage across 64 peptides
25
75
125
175
225
275
325
Residue Number
BamD
86.7 % coverage across 62 peptides
20
70
120
170
220
Residue Number
BamE
99.0 % coverage across 20 peptides
20
30
40
50
60
70
80
90
100
110
120
Residue Number
SurA
83.9 % coverage across 64 peptides
20
70
120
170
220
270
320
370
420
Residue Number

Figure S1. Sequence coverage maps of BAM subunits and SurA in HDX-MS experiments. Thick bars at the top of each panel represent sequence coverage with regions shaded in blue or grey indicating regions that were covered or not covered by detected peptides, respectively. Small blue bars represent the individual peptides detected.

BamA

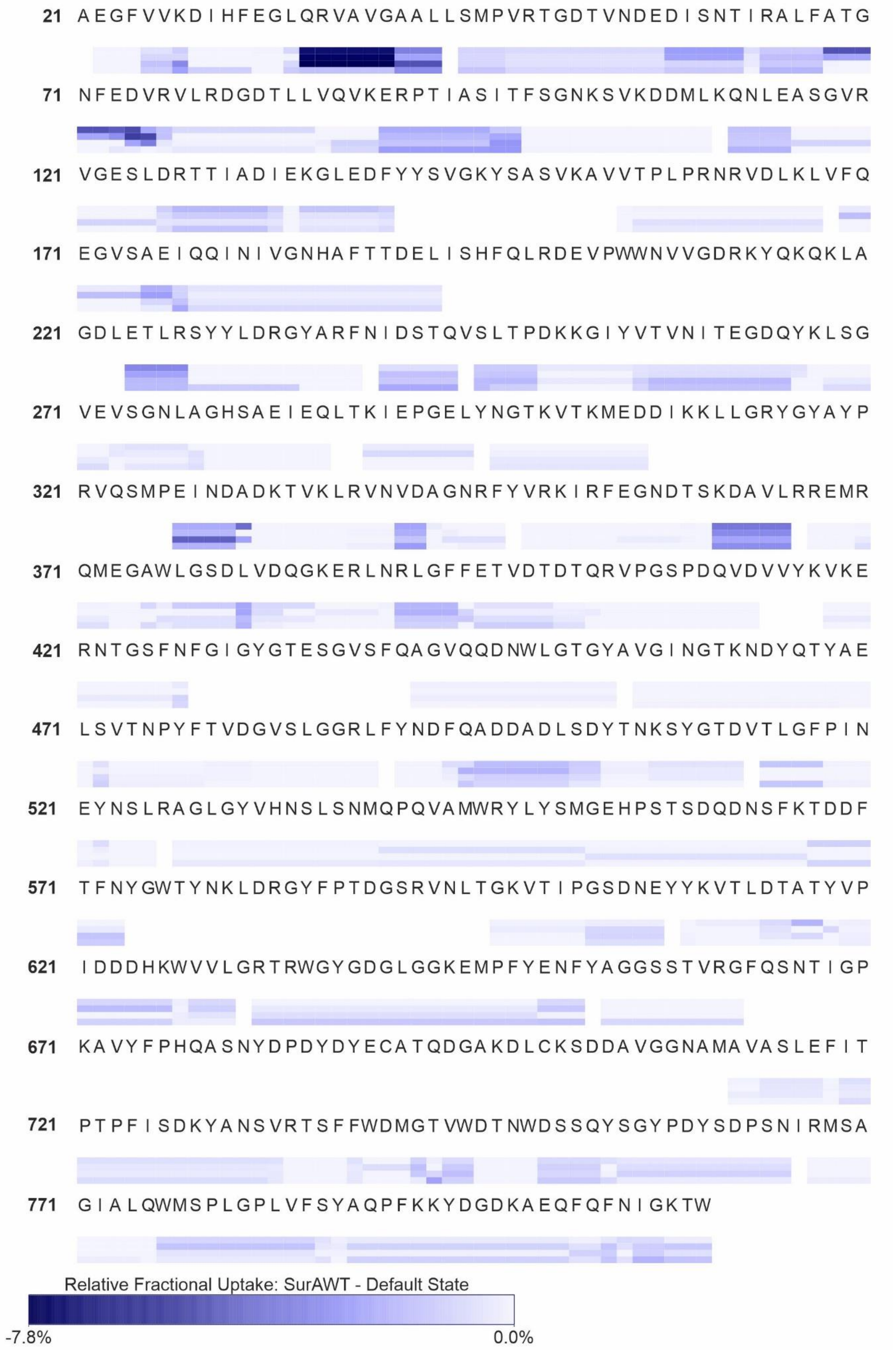
21 AEGFVVKDIHFEGLQRVAVGAALLSMPVRTGDTVNDEDISNTIRALFATG
71 NFEDVRVLRDGDTLLVQVKERPTIASITFSGNKSVKDDMLKQNLEASGVR
121 VGESLDRTTIADIEKGLEDFYYSVGKYSASVKAVVTPLPRNRVDLKLVFQ
171 EGVSAEIQQINIVGNHAFTTDELISHFQLRDEVPWWNVVGDRKYQKQKLA
221 GDLETLRSYYLDRGYARFNIDSTQVSLTPDKKGIYVTVNITEGDQYKLSG
271 VEVSGNLAGHSAEIEQLTKIEPGELYNGTKVTKMEDDIKKLLGRYGYAYP
321 RVQSMPEINDADKTVKLRVNVDAGNRFYVRKIRFEGNDTSKDAVLRREMR
371 QMEGAWLGSDLVDQGKERLNRLGFFETVDTDTQRVPGSPDQVDVVYKVKE
421 RNTGSFNFGIGYGTESGVSFQAGVQQDNWLGTGYAVGINGTKNDYQTYAE
471 LSVTNPYFTVDGVSLGGRLFYNDFQADDADLSDYTNKSYGTDVTLGFPIN
521 EYNSLRAGLGYVHNSLSNMQPQVAMWRYLYSMGEHPSTSDQDNSFKTDDF
571 TFNYGWTYNKLDRGYFPTDGSRVNLTGKVTIPGSDNEYYKVTLDTATYVP
621 IDDDHKWVVLGRTRWGYGDGLGGKEMPFYENFYAGGSSTVRGFQSNTIGP
671 KAVYFPHQASNYDPDYDYECATQDGAKDLCKSDDAVGGNAMAVASLEFIT
721 PTPFISDKYANSVRTSFFWDMGTVWDTNWDSSQYSGYPDYSDPSNIRMSA
771 GIALQWMSPLGPLVFSYAQPFKKYDGDKAEQFQFNIGKTW
Relative Fractional Uptake: SurAWT - Default State
-7.8%
0.0%

BamB

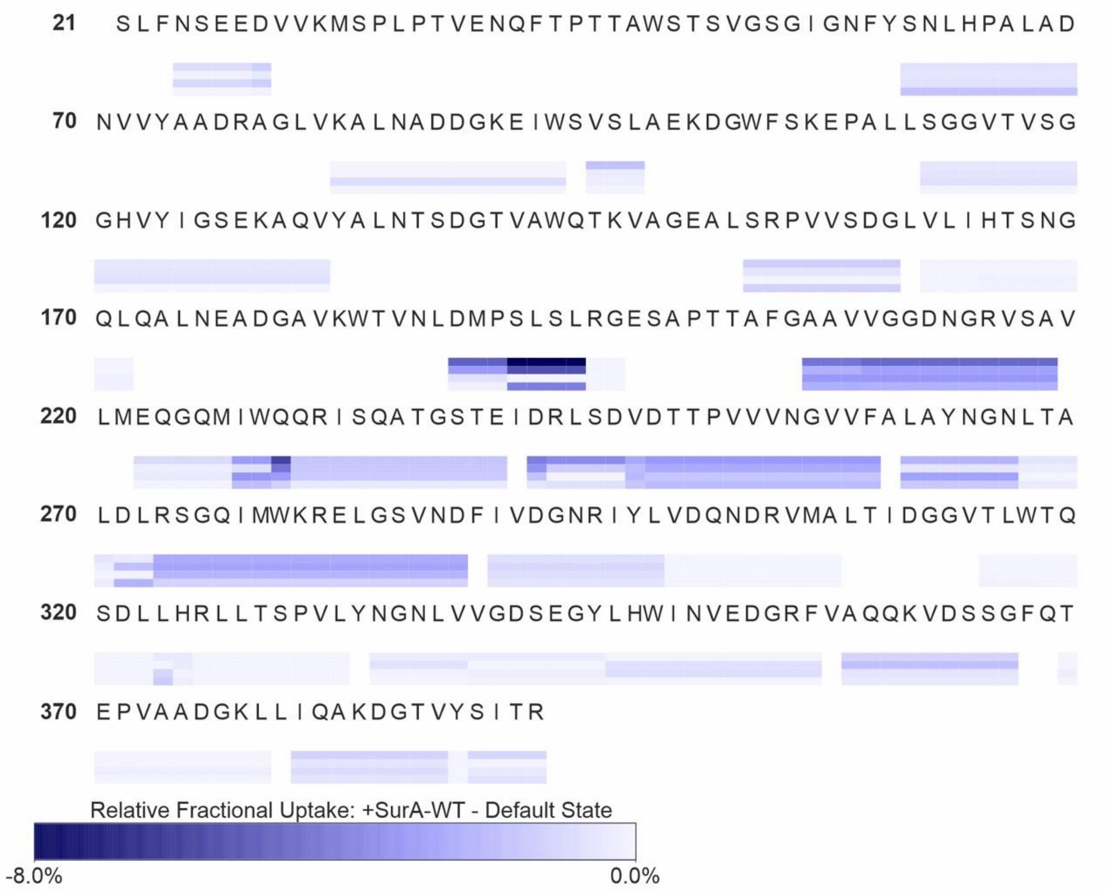
21 SLFNSEEDVVKMSPLPTVENQFTPTTAWSTSVGSGIGNFYSNLHPALAD
70 NVVYAADRAGLVKALNADDGKEIWSVSLAEKDGWFSKEPALLSGGVTVSG
120 GHVYIGSEKAQVYALNTSDGTVAWQTKVAGEALSRPVVSDGLVLIHTSNG
170 QLQALNEADGAVKWTVNLDMPSLSLRGESAPTTAFGAAVVGGDNGRVSAV
220 LMEQGQMIWQQRISQATGSTEIDRLSDVDTTPVVVNGVVFALAYNGNLTA
270 LDLRSGQIMWKRELGSVNDFIVDGNRIYLVDQNDRVMALTIDGGVTLWTQ
320 SDLLHRLLTSPVLYNGNLVVGDSEGYLHWINVEDGRFVAQQKVDSSGFQT
370 EPVAADGKLLIQAKDGTVYSITR
Relative Fractional Uptake: +SurA-WT - Default State
-8.0%
0.0%

BamC

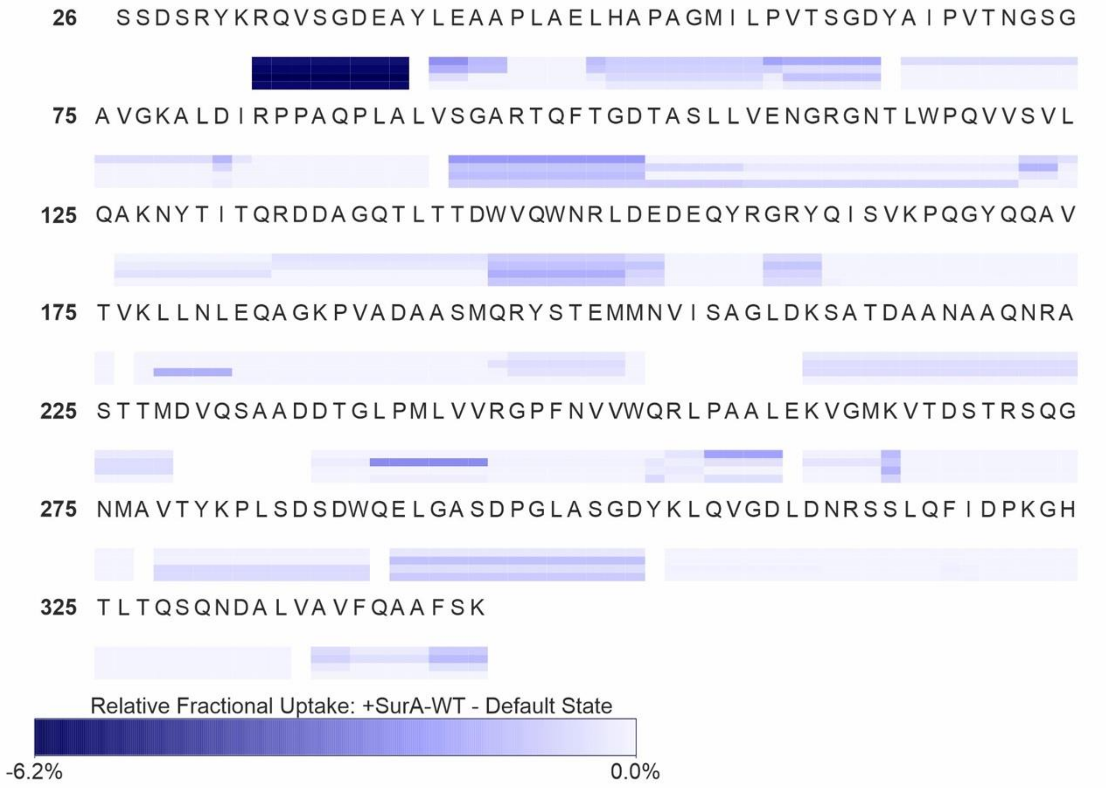
26 SSDSRYKRQVSGDEAYLEAAPLAELHAPAGMILPVTSGDYAIPVTNGSG
75 AVGKALDIRPPAQPLALVSGARTQFTGDTASLLVENGRGNTLWPQVVSVL
125 QAKNYTITQRDDAGQTLTTDWVQWNRLDEDEQYRGRYQISVKPQGYQQAV
175 TVKLLNLEQAGKPVADAASMQRYSTEMMNVISAGLDKSATDAANAAQNRA
225 STTMDVQSAADDTGLPMLVVRGPFNVVWQRLPAALEKVGMKVTDSTRSQG
275 NMAVTYKPLSDSDWQELGASDPGLASGDYKLQVGDLDNRSSLQFIDPKGH
325 TLTQSQNDALVAVFQAAFSK
Relative Fractional Uptake: +SurA-WT - Default State
-6.2%
0.0%

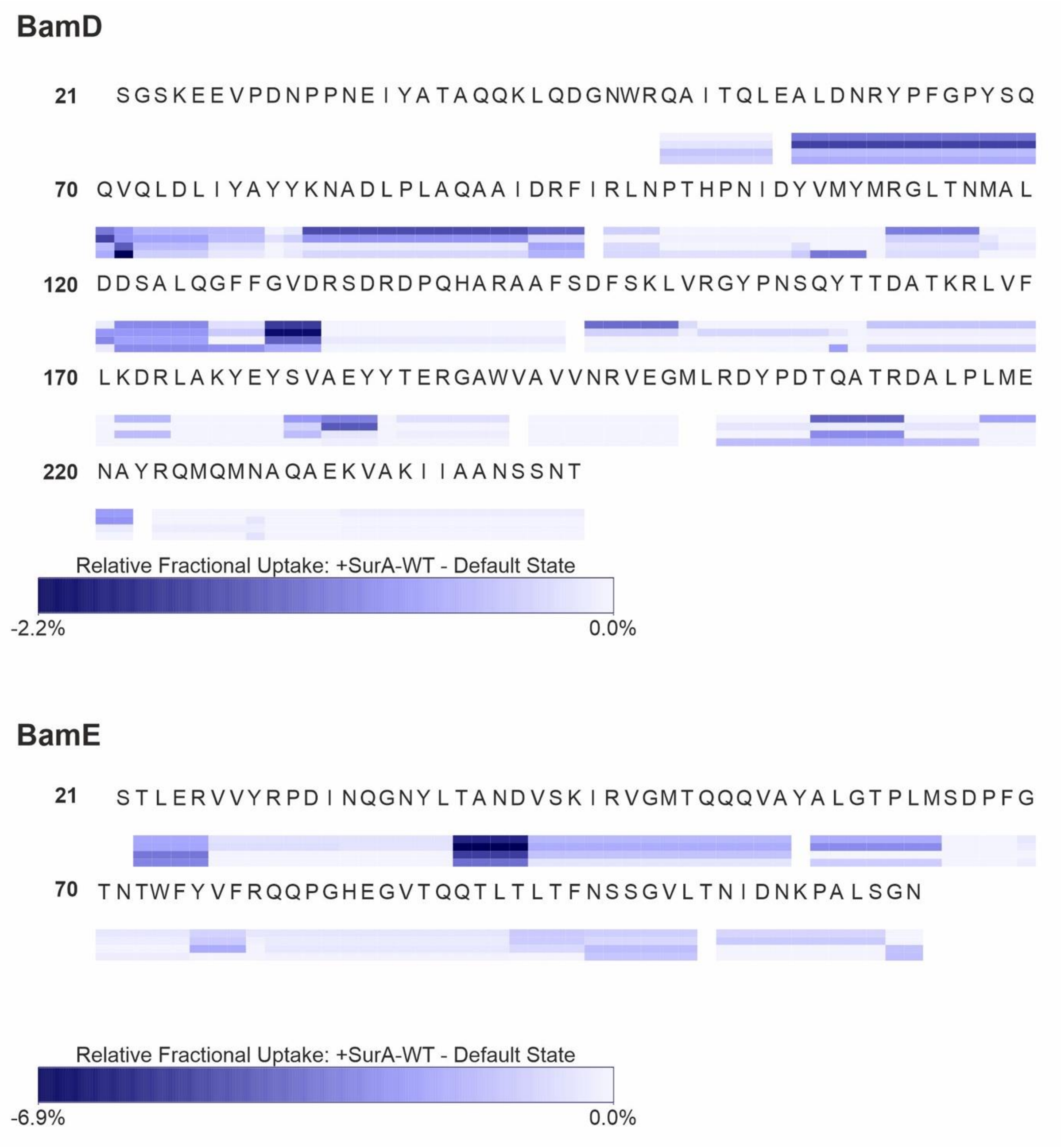

Figure S2. Difference in relative fraction uptake on a pseudo-residue level calculated using DynamX (Waters), comparing the extent of deuterium uptake in BAM subunits in the absence or presence of SurA-WT. The pseudo-residue level data shown here are obtained by only considering the shortest peptide that contains each residue and ignoring uptake data from any other overlapping peptides.

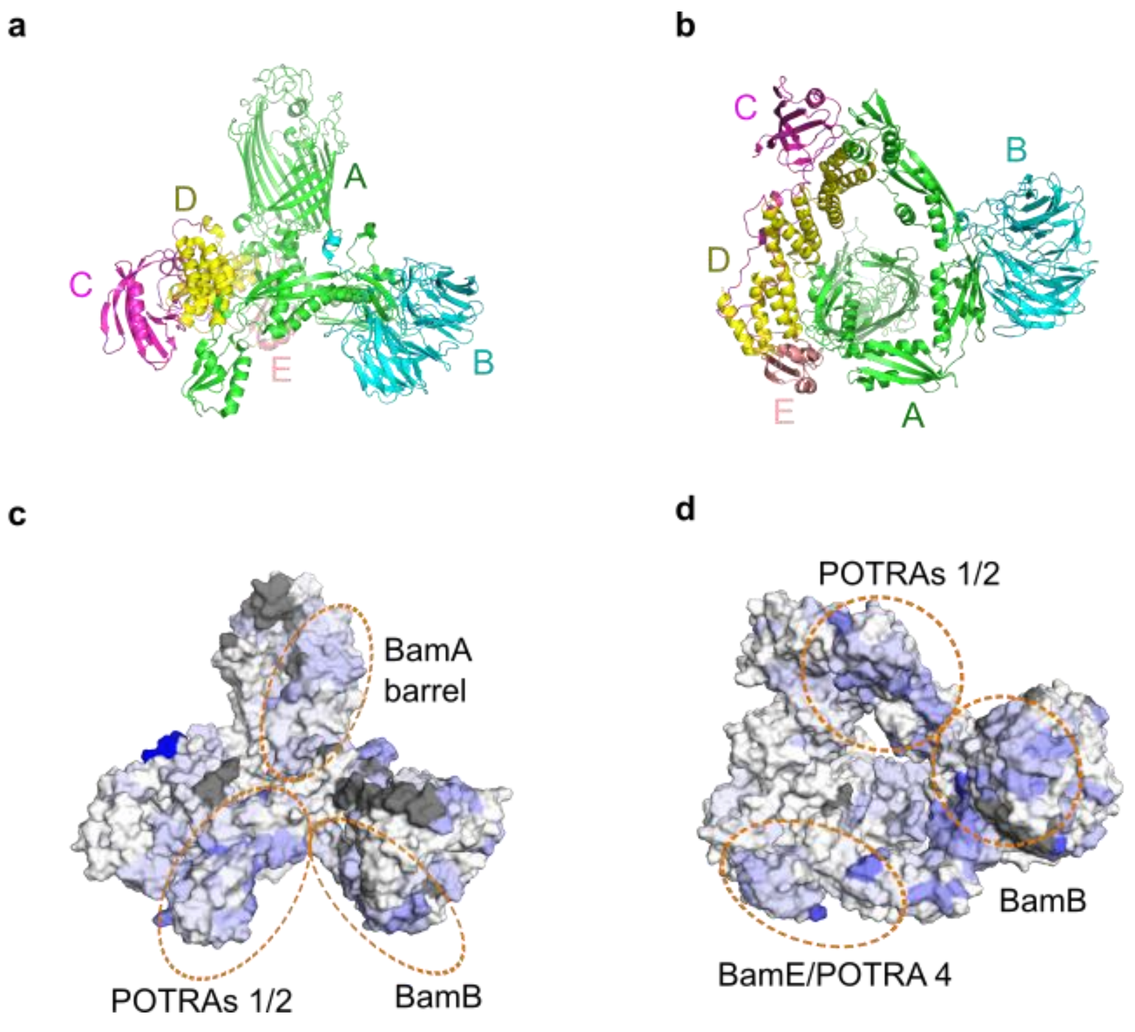

Figure S3. Pseudo-residue level HDX-MS analysis of BAM in the presence of SurA. (**a,b**) The structure of the BAM complex in the 'lateral-open' conformation (PDB: 5LJO[1]) coloured by subunit. (**c,d**) surface views showing pseudo-residue level HDX protection in the BAM complex upon binding SurA. Left panels show a side view of the complex, and right panels a view from the periplasmic face. Regions in dark grey denote sequences for which peptides were not detected. Protection levels for each residue are shown scaled from white to blue where white indicates no protection and blue corresponds to the maximum observed protection. Data for the 2 hr HDX timepoint is shown, and the pseudo-residue level data was obtained by considering the shortest peptide that contains each residue and ignoring uptake data from any other overlapping peptides. Patches of protection from HDX upon SurA binding (**Fig. 2**) in the BamA β-barrel domain (adjacent to the lateral gate), POTRAs 1 and 2, BamB, and BamE/POTRA 4 are ringed in orange.

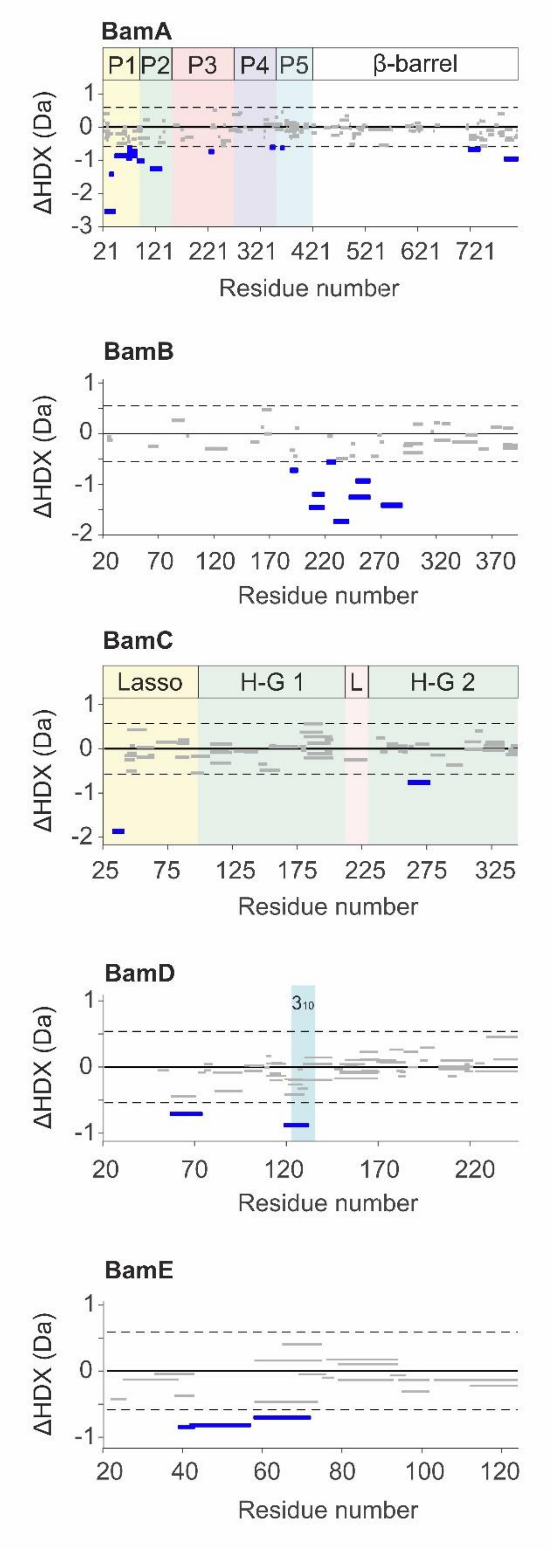

Figure S4. Woods plots showing the summed differences in deuterium uptake in BAM subunits over all four HDX timepoints (0.5, 2, 30 and 120 min of HDX), comparing BAM alone with BAM in the presence of SurA-WT. Woods plots were generated using Deuteros[2]. Peptides coloured in blue are protected from exchange in the presence of SurA. Peptides with no

significant difference between conditions, determined using a 99% confidence interval (dotted line), are shown in grey. See Methods for experimental details. The locations of the five POTRA domains (P1-P5) and the β-barrel domain of BamA are indicated. For BamC, the ‘lasso’ domain and the two helix-grip domains (HG-1 and HG-2) separated by a linker (L) are highlighted. The location of the 3_{10} helix in BamD is also indicated, as these motifs are mentioned specifically in the main text.

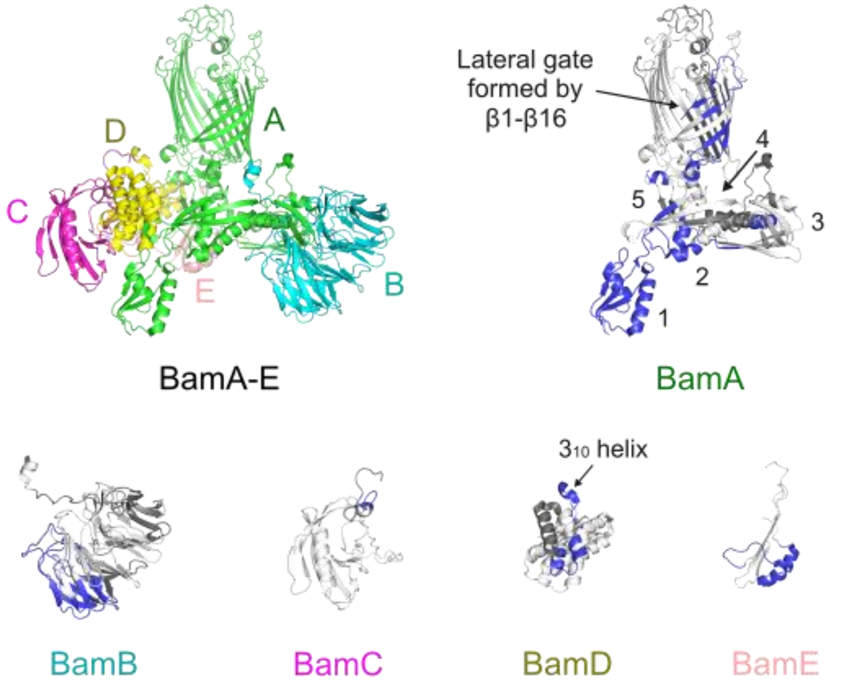

Figure S5. Subunits of BAM showing regions of protection from HDX in the presence of SurA-WT. The structure of the BAM complex coloured by subunit (top left) and BAM subunits coloured to show the HDX-MS data. Regions in blue show protection from exchange in the presence of SurA. Note that there were no detected regions of deprotection. Regions in white show no change in deuterium uptake in the presence of SurA. Regions for which peptides were not detected are highlighted in dark grey. The lateral gate and five POTRA domains in BamA are indicated, as is the 3_{10} helix in BamD, as these motifs are mentioned specifically in the main text.

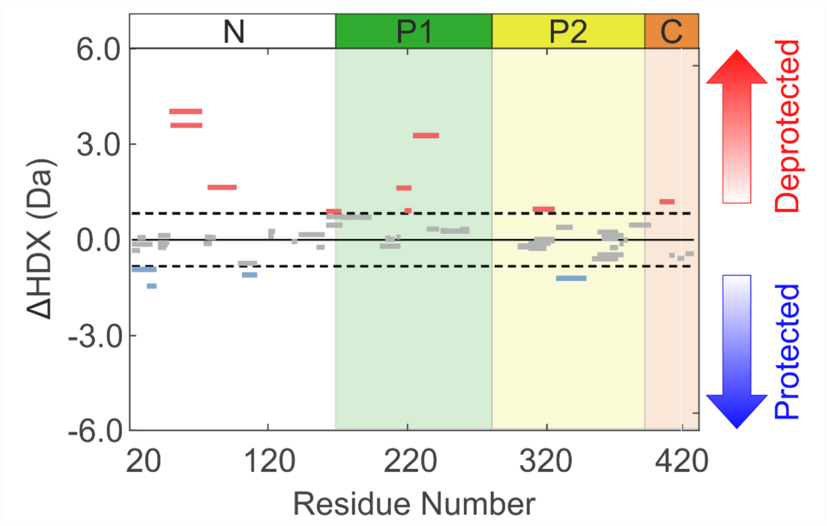

Figure S6. Woods plot showing the summed differences in deuterium uptake in SurA over all four HDX timepoints (0.5, 2, 30 and 120 min of HDX), comparing SurA alone with SurA in the presence of BAM. The Woods plot was generated using Deuteros[2]. Peptides coloured in blue are protected from exchange in the presence of BAM. Peptides with no significant difference between conditions, determined using a 99% confidence interval (dotted line), are shown in grey. See Methods for experimental details.

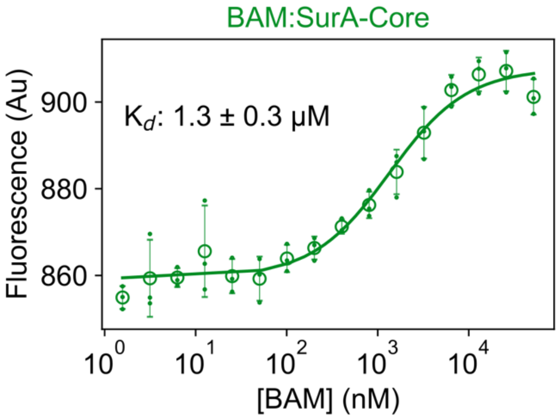

Figure S7. Microscale thermophoresis (MST) data for binding of SurA-core to BAM in DDM. Samples contained 400 nM Alexa Fluor 488-labelled SurA-core (see Methods), BAM (1.6 nM - 26 μM), 0.02 % (*v/v*) DDM, 150 mM NaCl, 20 mM Tris-HCl, pH 8, at 25 °C. Three independent replicates were performed and averaged prior to fitting. The mean for each BAM concentration is shown as open circles and the individual values for each replicate are shown as dots. The error bars represent the standard deviation between replicates. Data were fitted to a 1:1 quadratic binding model (see Methods). Source data are provided as a Source Data file (**Supplementary Data 8**).

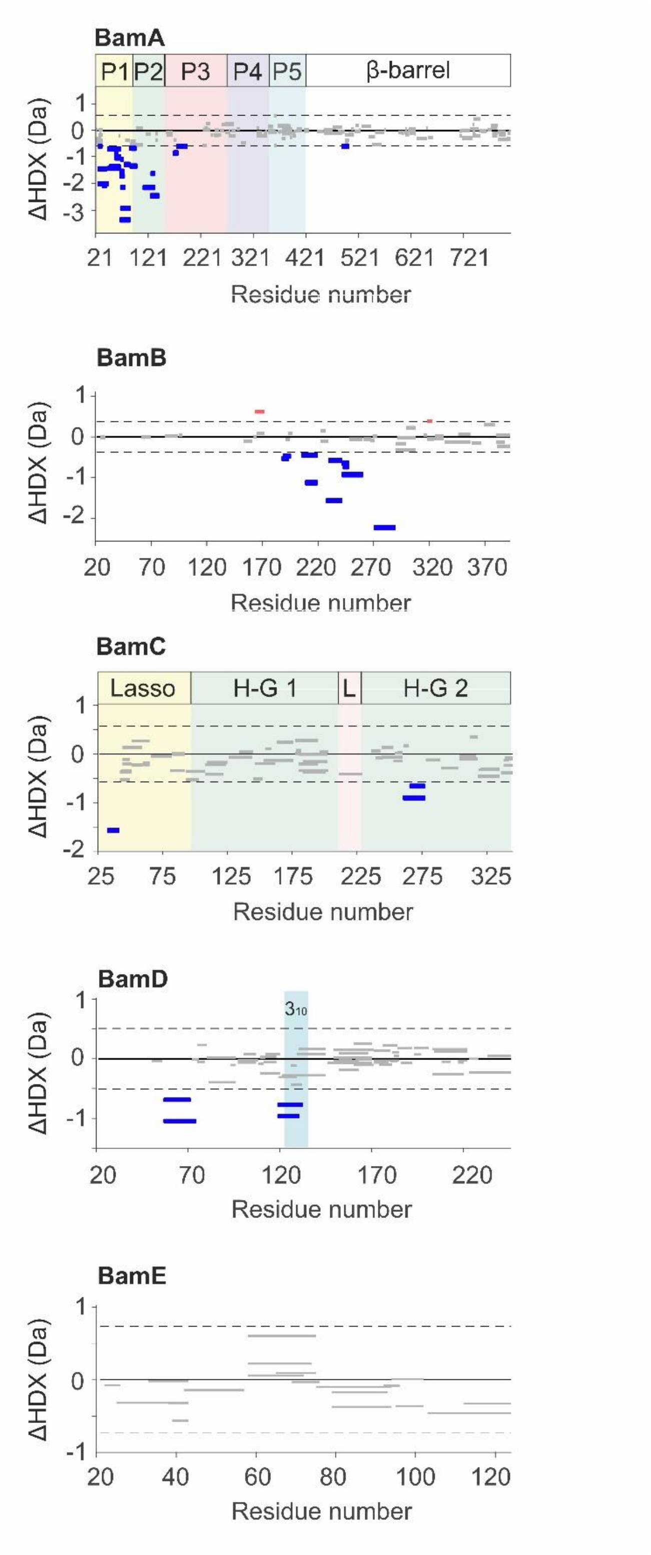

Figure S8. Woods plots showing the summed differences in deuterium uptake in BAM subunits over all four HDX timepoints, comparing BAM alone with BAM in the presence of SurA-core. Woods plots were generated using Deuteros[2]. Peptides coloured in blue are protected from exchange in the presence of SurA-core. Peptides with no significant difference between conditions, determined using a 99% confidence interval (dotted line), are shown in

grey. See Methods for experimental details. The locations of the five POTRA domains (P1-P5) and β-barrel domain of BamA are indicated. In BamC, the 'lasso' domain and the two helix-grip domains (HG-1 and HG-2) separated by a linker (L) are indicated. The location of the 3_{10} helix in BamD is also indicated.

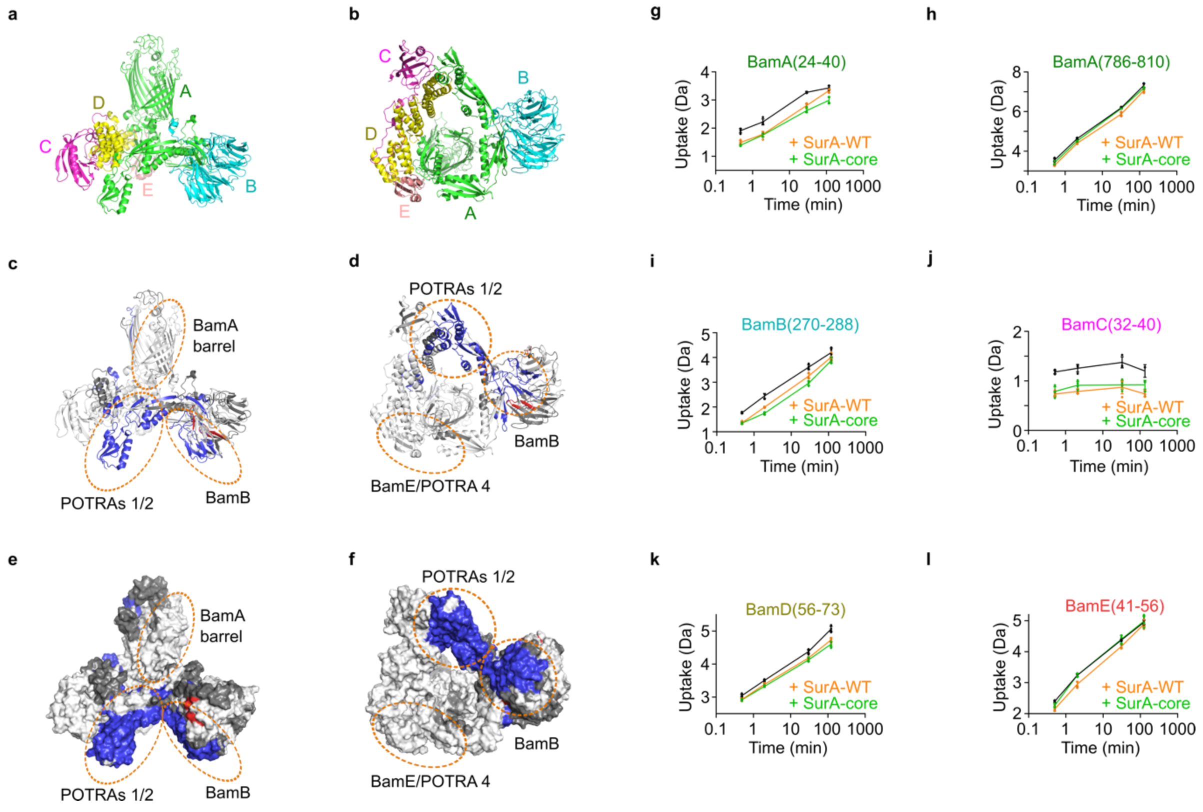

Figure S9. HDX-MS analysis of BAM in the presence of SurA-core. (**a,b**) The structure of the BAM complex in the 'lateral-open' conformation (PDB: 5LJO[1]) coloured by subunit. (**c,d**) Cartoon view and (**e,f**) surface views showing regions of HDX protection in the BAM complex upon binding SurA-core. Left panels show a side view of the complex, and right panels a view from the periplasmic face. Regions that are protected or deprotected from hydrogen exchange in the presence of SurA are highlighted in blue or red, respectively. Regions in white show no change in deuterium uptake in the presence of SurA, while those in dark grey denote sequences for which peptides were not detected. Note that one small region in BamB showed deprotection from HDX upon SurA binding. Regions for which patches of protection from HDX were observed upon binding of SurA-WT binding are ringed in orange. Compared with the HDX data for BAM in complex with SurA-WT (**Fig. 2**), similar protection is observed for SurA-core binding to BAM in POTRAs 1 and 2 and BamB. However, for SurA-core binding to BAM there is no protection in the BamE/POTRA 4 region, consistent with this region interacting with the SurA P2 domain. In addition, there is no protection in the BamA β-barrel domain (adjacent to the lateral gate), suggesting that SurA-core does not trigger the same conformational changes in BAM (which may prime it to accept substrates), or at least not to the same extent as observed for SurA-WT. (**g-l)** Representative deuterium uptake plots for peptides from (**g,h**) BamA, (**i**) BamB, (**j**) BamC, (**k**) BamD, and (**l**) BamE. The extent of deuterium uptake (Da) in the absence (black) and presence of SurA-core (green) or SurA-WT (orange) is shown.

Individual data points for each time point are shown as dots and error bars represent the standard deviation of three technical replicates. Source data are provided as a Source Data file (**Supplementary Data 9**).

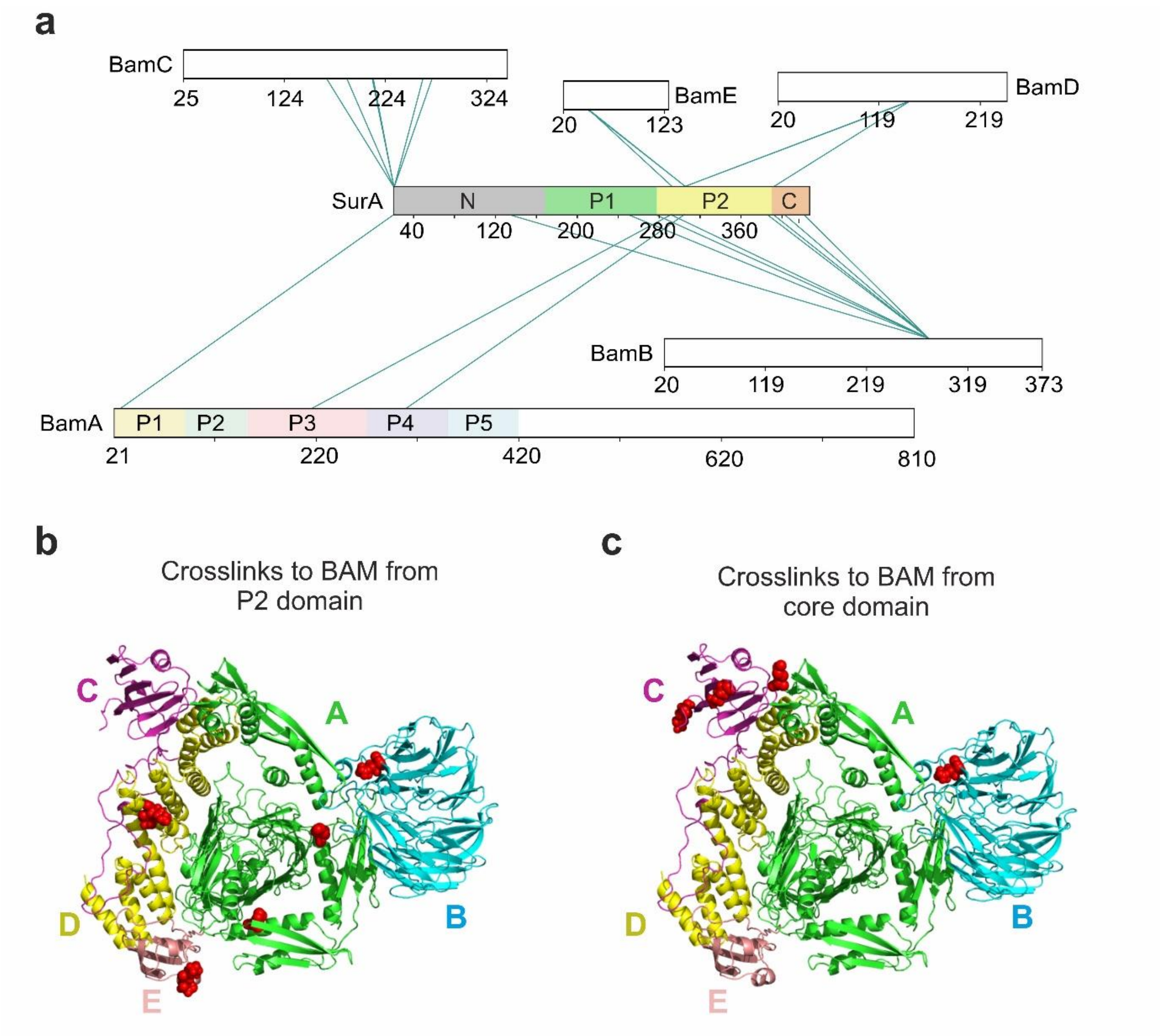

Figure S10. Crosslinks detected between SurA and the BAM complex using the homobifunctional NHS-ester crosslinker DSBU. (**a**) Map showing all detected inter-protein crosslinks involving SurA-WT. SurA is coloured according to its domain structure (see Figure 1 in the main text). The 5 POTRA domains (P1-P5) of BamA are indicated. (**b**) Residues on BAM that are involved in crosslinks with the P2 domain of SurA are shown as red spheres. (**c**) Residues on BAM that are involved in crosslinks with the core domain of SurA are shown as red spheres. A list of detected crosslinks can be found in **Table S1**.

a

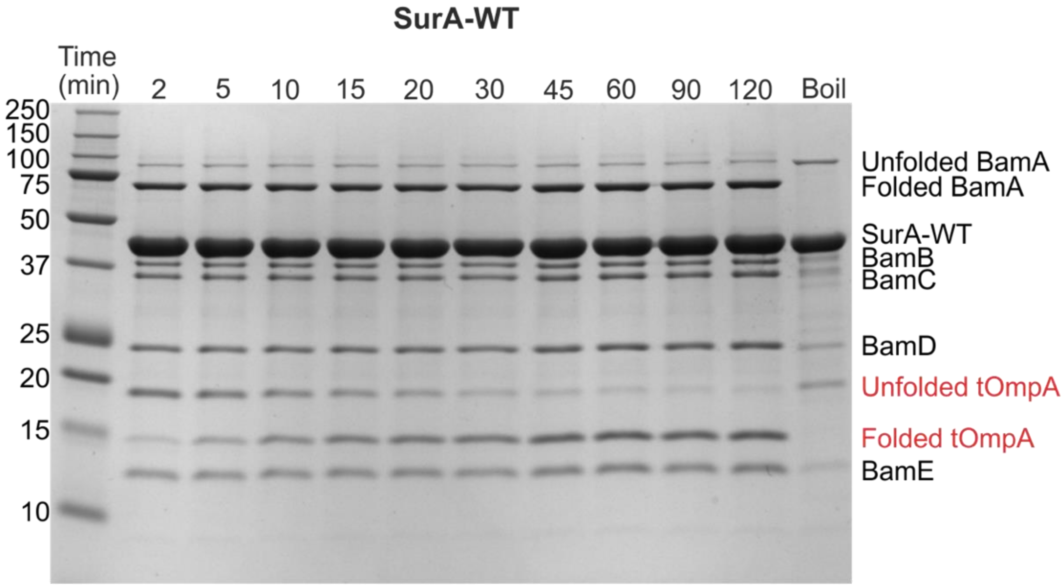
SurA-WT
Time (min)
2
5
10
15
20
30
45
60
90
120
Boil
250
150
100
75
50
37
25
20
15
10
Unfolded BamA
Folded BamA
SurA-WT
BamB
BamC
BamD
Unfolded tOmpA
Folded tOmpA
BamE

b

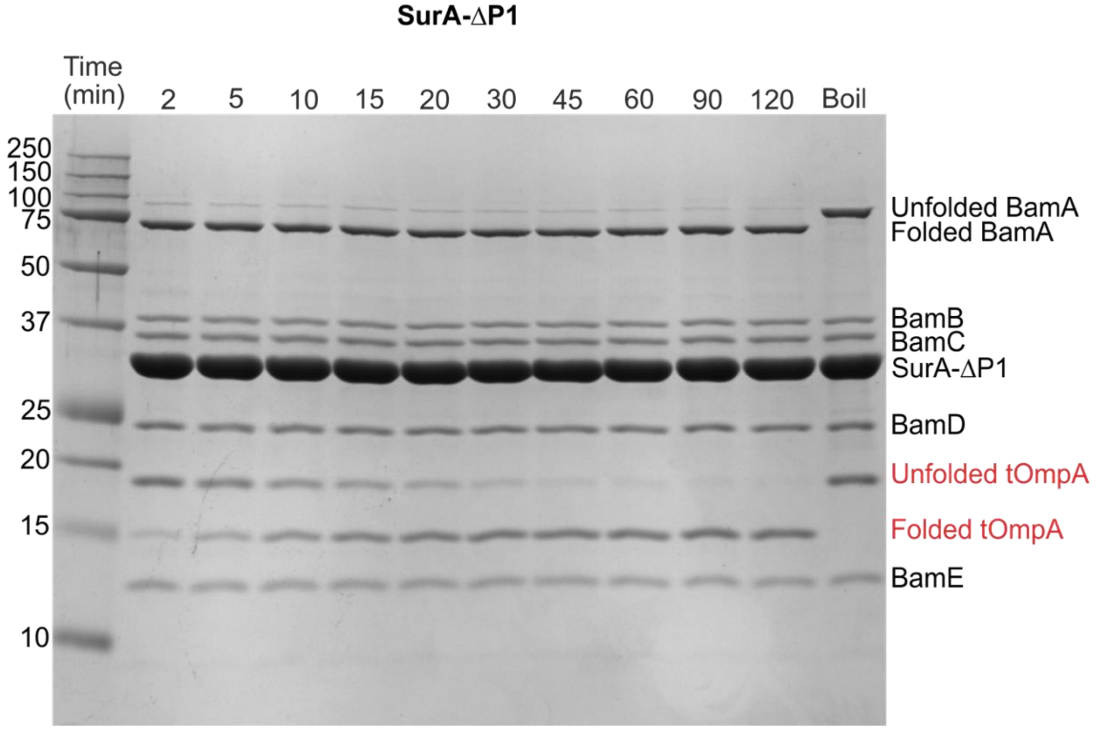
SurA-ΔP1
Time (min)
2
5
10
15
20
30
45
60
90
120
Boil
250
150
100
75
50
37
25
20
15
10
Unfolded BamA
Folded BamA
BamB
BamC
SurA-ΔP1
BamD
Unfolded tOmpA
Folded tOmpA
BamE

c

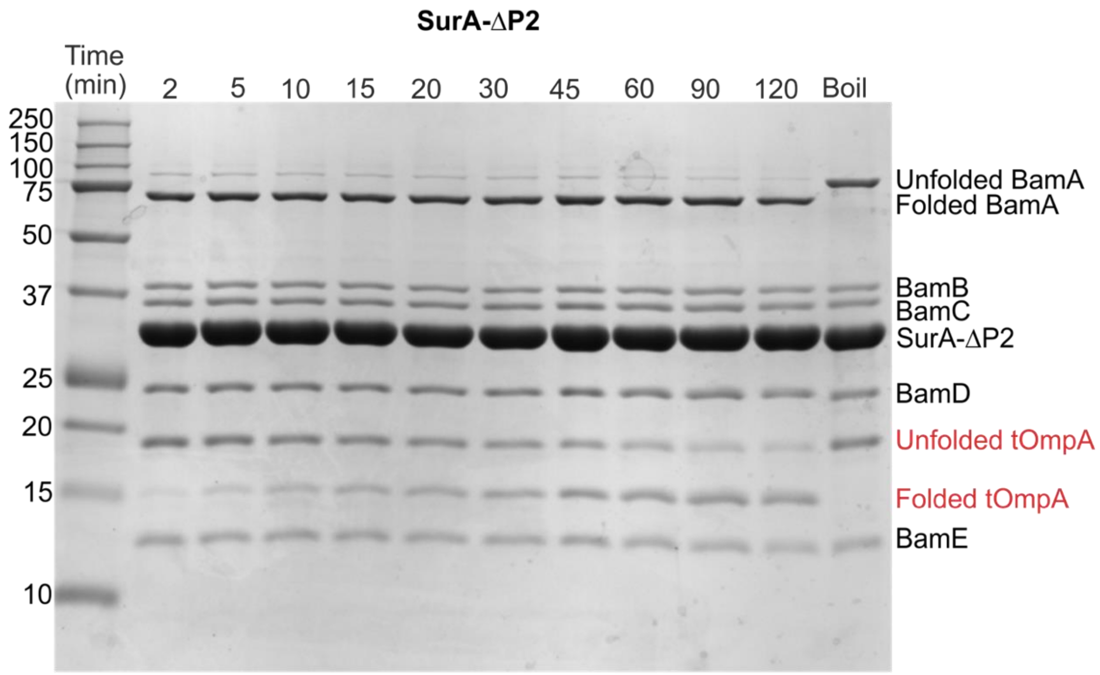
SurA-∆P2
Time (min)
2
5
10
15
20
30
45
60
90
120
Boil
250
150
100
75
50
37
25
20
15
10
Unfolded BamA
Folded BamA
BamB
BamC
SurA-∆P2
BamD
Unfolded tOmpA
Folded tOmpA
BamE

d

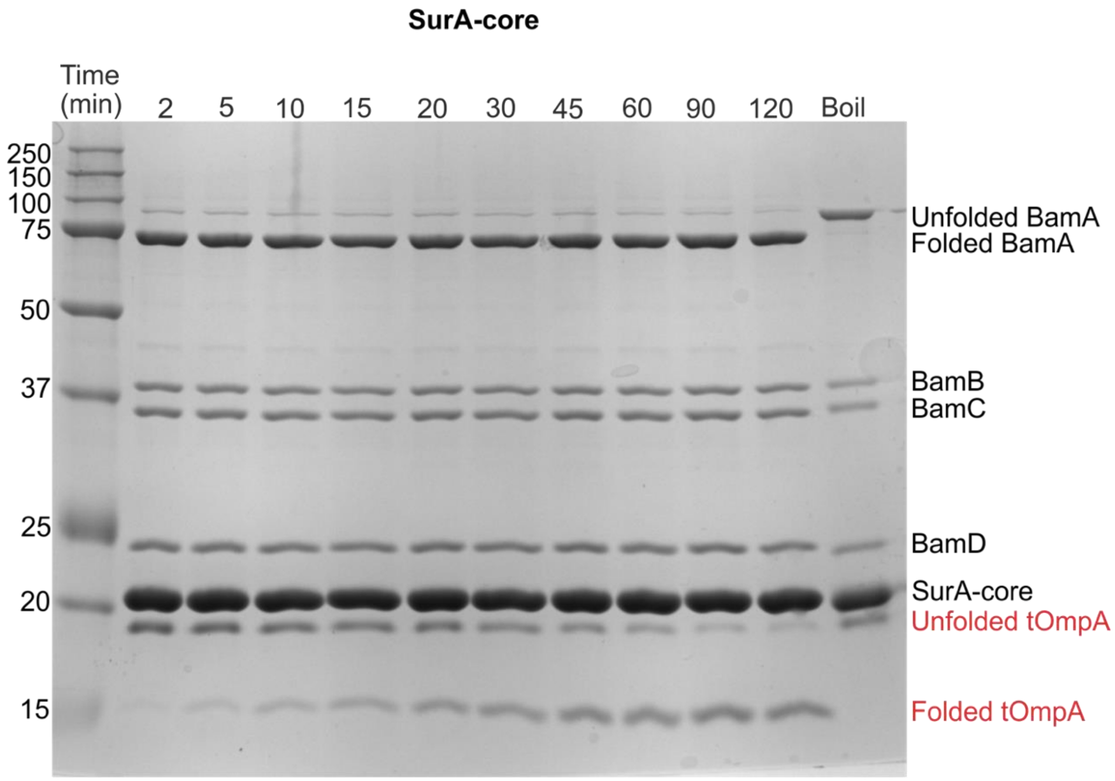
SurA-core
Time (min)
2
5
10
15
20
30
45
60
90
120
Boil
250
150
100
75
50
37
25
20
15
Unfolded BamA
Folded BamA
BamB
BamC
BamD
SurA-core
Unfolded tOmpA
Folded tOmpA

e

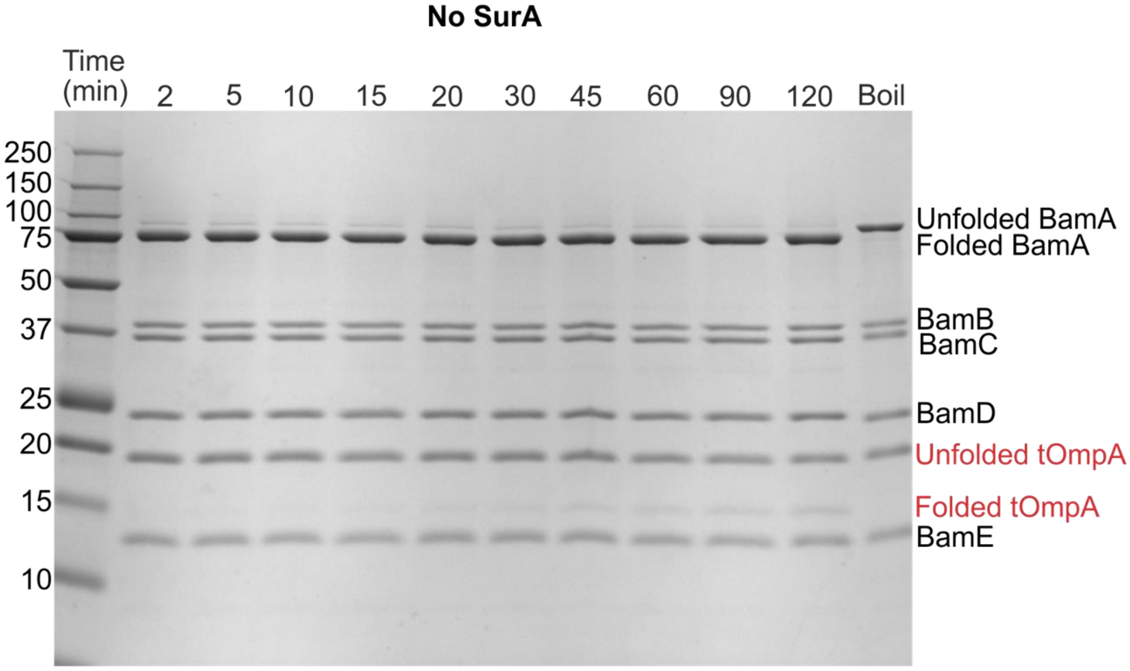
No SurA
Time (min)
2
5
10
15
20
30
45
60
90
120
Boil
250
150
100
75
50
37
25
20
15
10
Unfolded BamA
Folded BamA
BamB
BamC
BamD
Unfolded tOmpA
Folded tOmpA
BamE

f

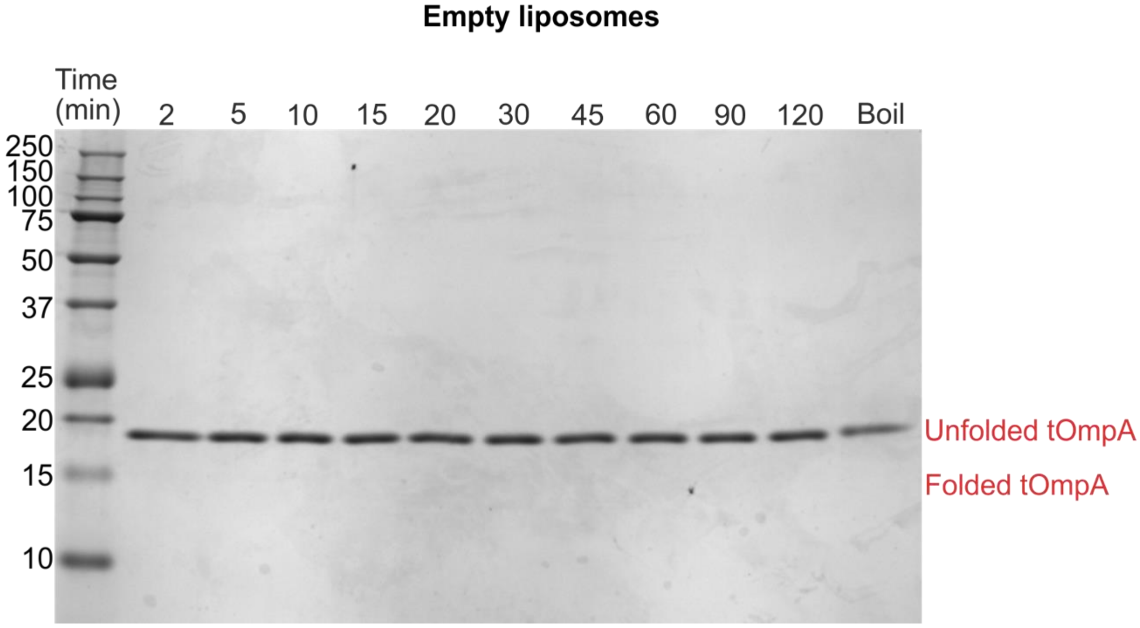
Empty liposomes
Time (min)
2
5
10
15
20
30
45
60
90
120
Boil
250
150
100
75
50
37
25
20
15
10
Unfolded tOmpA
Folded tOmpA

g

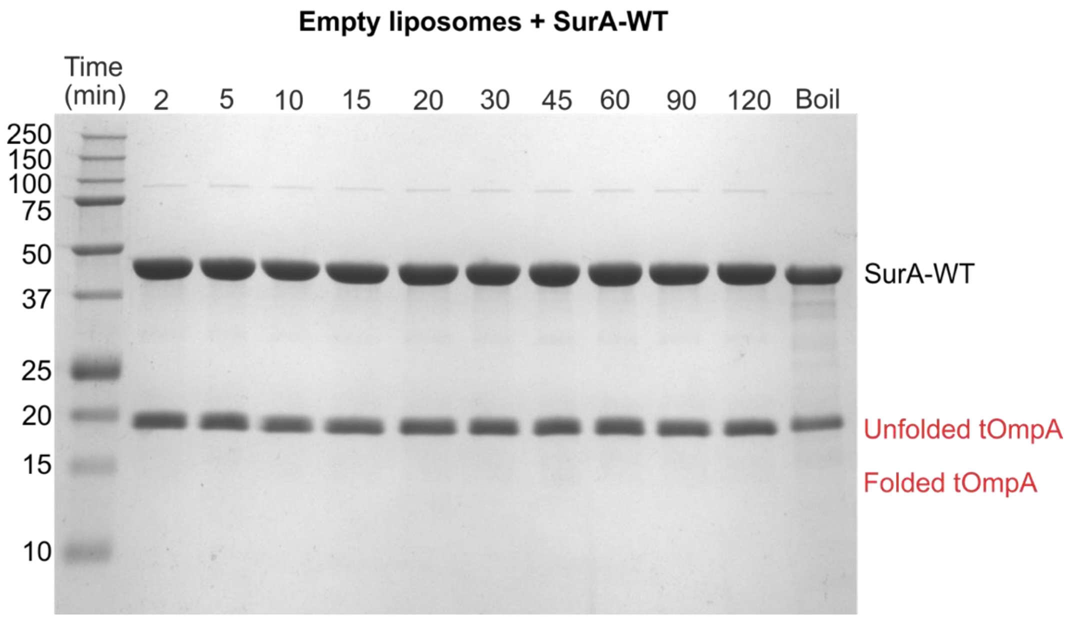

Figure S11. Example cold SDS-PAGE gels for kinetic folding assays shown in **Fig. 4** main text. (**a-g**) Example gels for tOmpA folding reactions containing (**a**) BAM + SurA-WT, (**b**) BAM + SurA-ΔP1, (**c**) BAM + SurA-ΔP2, (**d**) BAM + SurA-core, (**e**) BAM alone (no SurA), (**f**) Empty liposomes (no BAM and no SurA), and (**g**) Empty liposomes (no BAM) + SurA-WT. Samples contained 2 µM tOmpA, 1 µM BAM (where included), 10 µM SurA or SurA variant (where included), 0.8 M urea, 20 mM Tris-HCl, 150 mM NaCl, pH 8.0, at 25 ˚C. Note that in experiments involving SurA-core (d), gels were run for additional time to ensure separation between the bands for SurA-core and unfolded tOmpA. Source data are provided as a Source Data file (**Supplementary Data 10**).

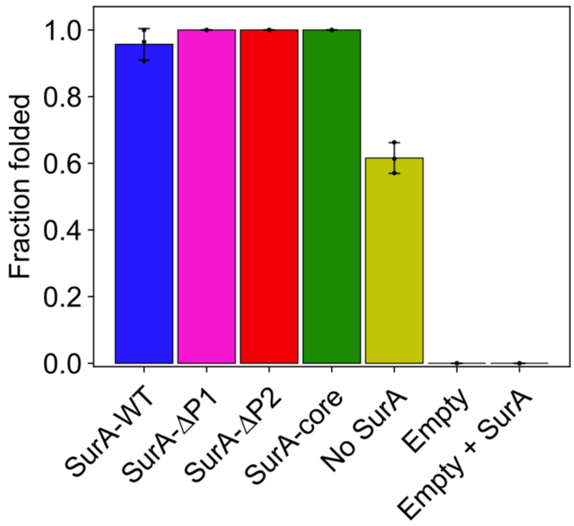

Figure S12. Overnight folding yields (~18 h) of tOmpA folding into BAM-containing proteoliposomes composed of *E. coli* polar lipid extract in the presence of different SurA domain deletion variants. Samples contained 2 µM tOmpA, 1 µM BAM (where included), 10 µM SurA or SurA variant (where included), 0.8 M urea, 20 mM Tris-HCl, 150 mM NaCl, pH 8.0, at 25 ˚C. Bars indicate the mean of three independent replicates, each using a different batch of BAM proteoliposomes. Error bars represent the standard deviation between replicates and individual data points are shown as dots. Empty: liposomes formed from *E. coli* polar lipid extract without the inclusion of BAM. Source data are provided as a Source Data file (**Supplementary Data 10**).

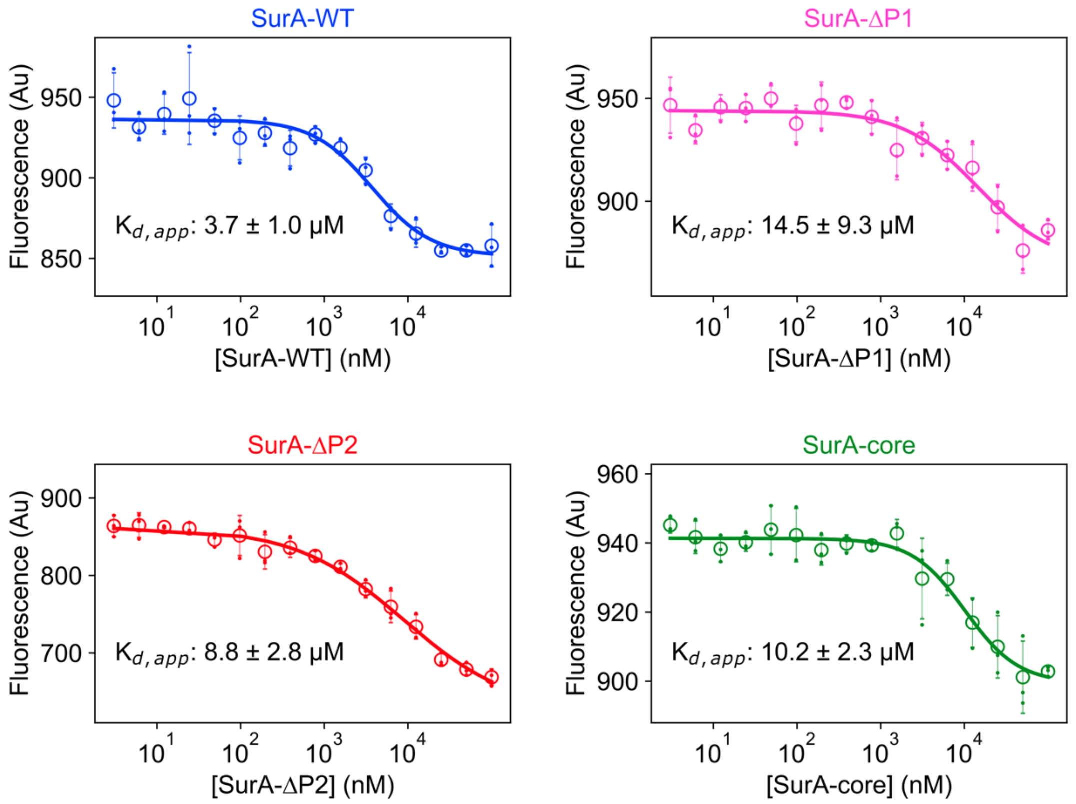

Figure S13. SurA-WT and its domain deletion variants have similar affinities for tOmpA. Samples for microscale thermophoresis (MST) contained 100 nM Alexa Fluor 488-labeled tOmpA, 100 µM-3 nM SurA or SurA domain deletion variant, 0.8 M urea, 20 mM Tris-HCl, pH 8.0, at 25 °C. Three independent replicates were performed and averaged prior to fitting. The mean for each SurA variant concentration is shown as open circles and the individual values for each replicate are shown as dots. The error bars represent the standard deviation between replicates. Data were fitted to a Hill binding model (see Methods). Source data are provided as a Source Data file (**Supplementary Data 8**).

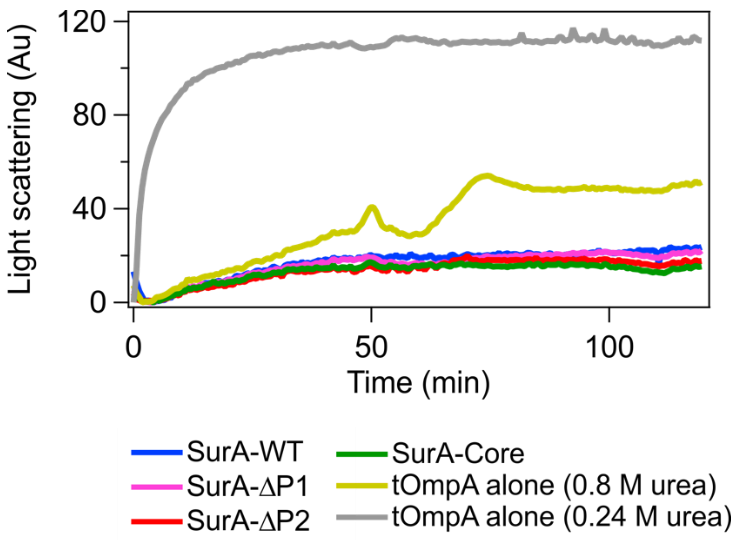

Figure S14. SurA-WT and domain deletion variants prevent aggregation of tOmpA under conditions employed in the kinetic refolding assays (Methods). Samples contained 2 μM tOmpA, 10 μM SurA variant, 0.8 M urea, 0.15 M NaCl and 20 mM Tris-HCl, pH 8.0. A control experiment at a lower urea concentration (0.24 M) and higher NaCl concentration (500 mM), which promotes aggregation[3], is shown for comparison (grey). Light scattering was measured by nephelometry at 635 nm for 2 h at 25 °C. Source data are provided as a Source Data file (**Supplementary Data 11**).

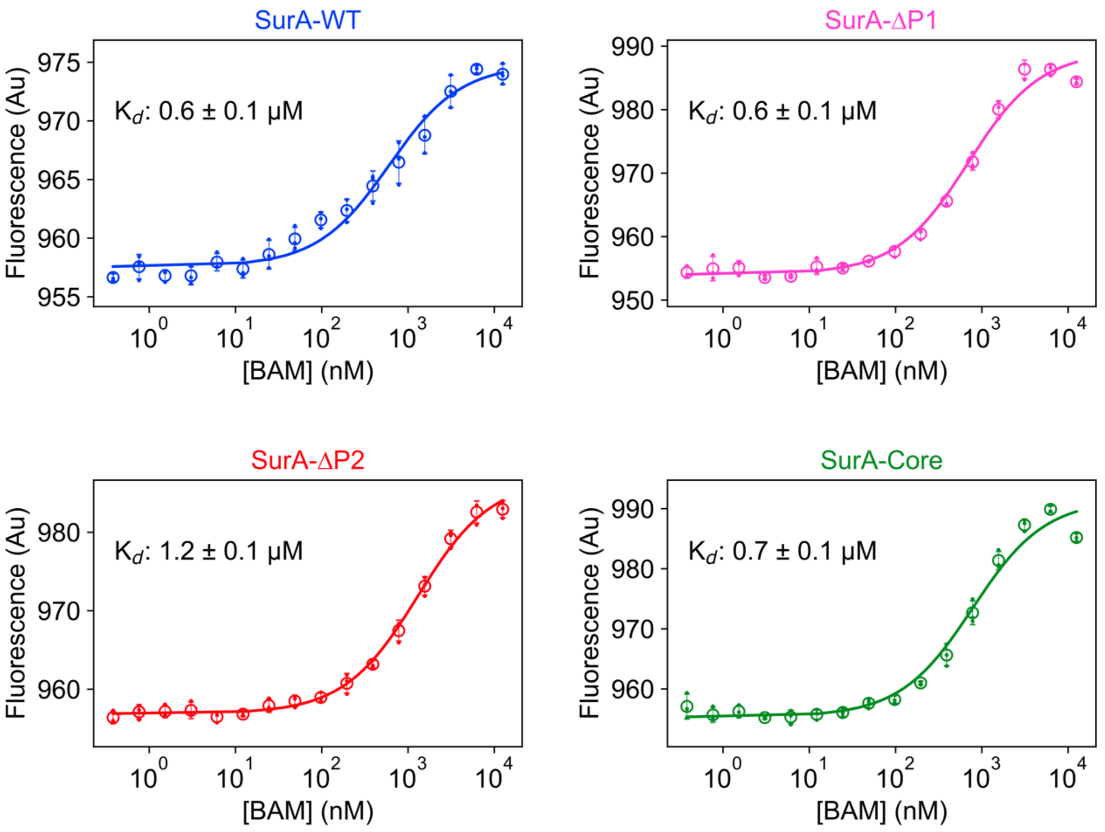

Figure S15. SurA-WT and its domain deletion variants bind BAM with similar affinity. Samples for microscale thermophoresis (MST) contained 400 nM Alexa Fluor 488-labeled SurA variant, 25 μM-1 nM BAM complex in proteoliposomes composed of *E. coli* polar lipid extract, 150 mM NaCl, 20 mM Tris-HCl, pH 8.0, at 25 °C. Previous studies have shown that reconstitution of BAM into liposomes results in an *ca.* equal population of 'inward' and 'outward' facing BAM complexes[4], we took the concentrations of BAM to be 50 % of the total in the sample for data fitting. Three independent replicates were performed and averaged prior to fitting. The mean for each BAM concentration is shown as open circles and the individual values for each replicate are shown as dots. The error bars represent the standard deviation between replicates. Data were fitted to a 1:1 quadratic binding model (see Methods). Source data are provided as a Source Data file (**Supplementary Data 8**).

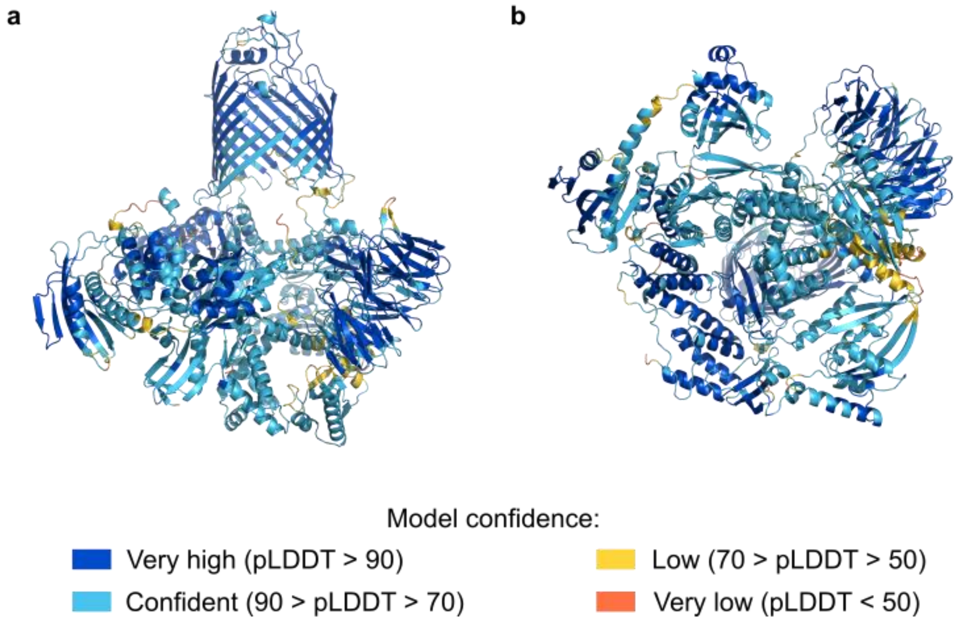

Figure S16. Predicted Local Distance Difference Test (pLDDT) scores for an AlphaFold-Multimer generated model of the BAM-SurA complex. Views of the BAM-SurA complex from **(a)** the side, and (**b**) the periplasmic face, coloured according to per residue model confidence as indicated. The high pLDDT scores indicate confidence in the prediction of local structural arrangement for most residues in both BAM and SurA.

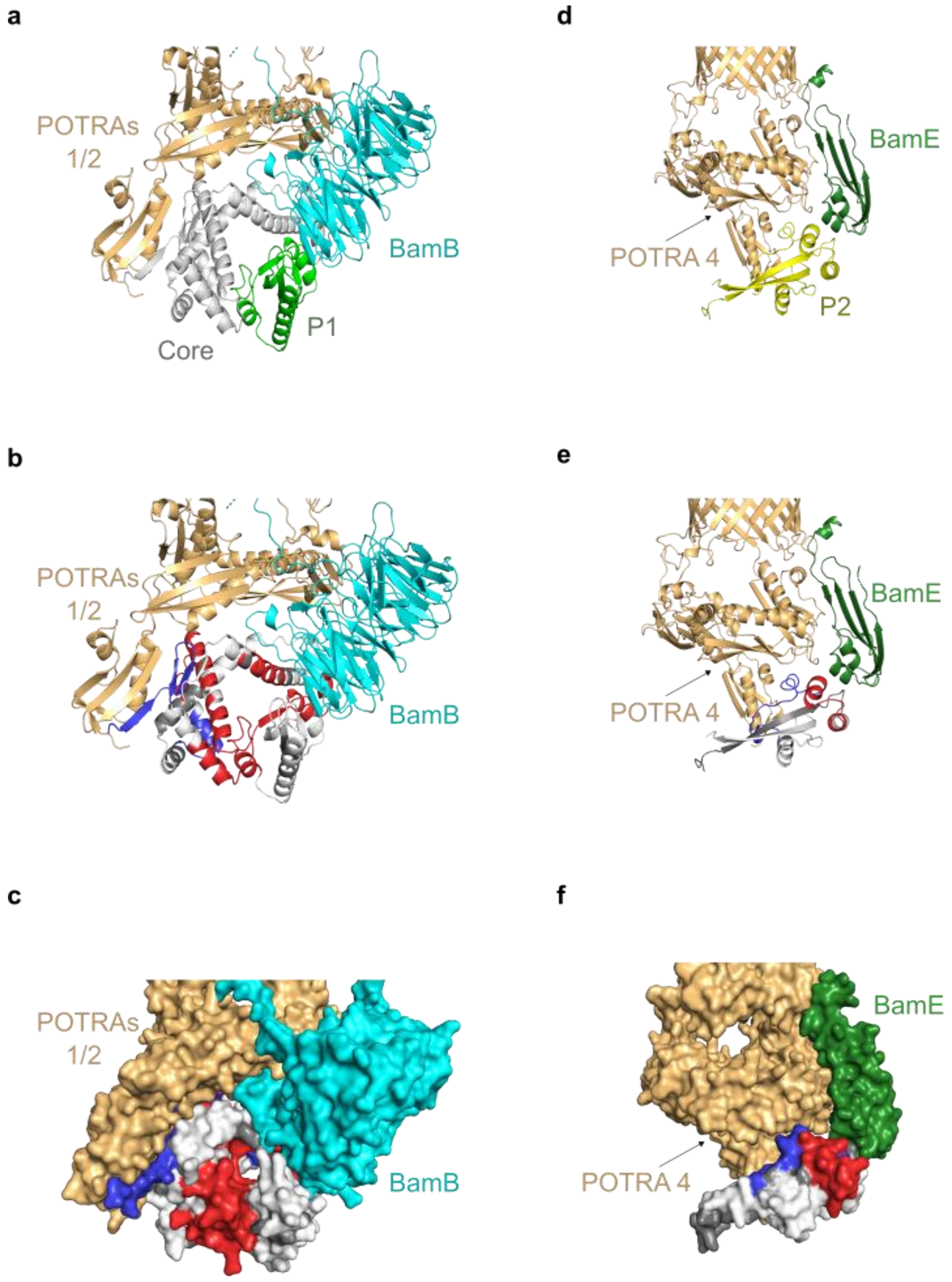

Figure S17. Comparison of the AlphaFold-Multimer predicted model of the BAM-SurA complex with HDX-MS data for SurA in the presence of BAM. **(a,b,c)** Predicted interactions of the SurA core and P1 domains with BamA POTRAs 1-2 and BamB shown as **(a)** cartoon view coloured by subunit, **(b,c)** cartoon and surface view, respectively, with protection and deprotection in SurA highlighted in blue and red, respectively. For clarity, BamC, BamD, BamE and the SurA P2 domain are not shown. **(d,e,f)** Predicted interactions of the SurA P2 domain with BamA POTRA 4 and BamE shown as **(d)** cartoon view coloured by subunit, **(e,f)** cartoon and surface view, respectively, with protection and deprotection in SurA highlighted in blue

and red, respectively. In **(b,c)** and **(e,f)** regions in white show no change in deuterium uptake in the presence of SurA, while those in grey denote sequences for which peptides were not detected. For clarity, BamB, BamC, BamD and the SurA P1 domain are not shown.

a

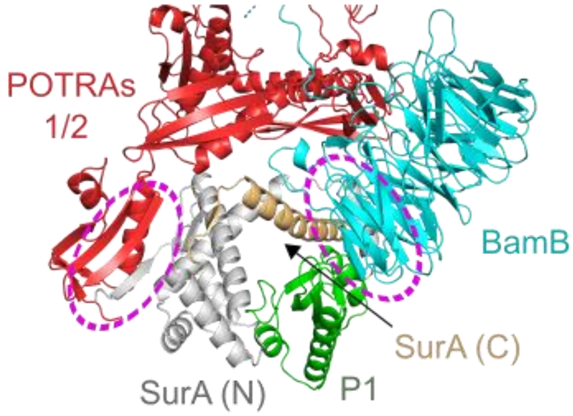

b

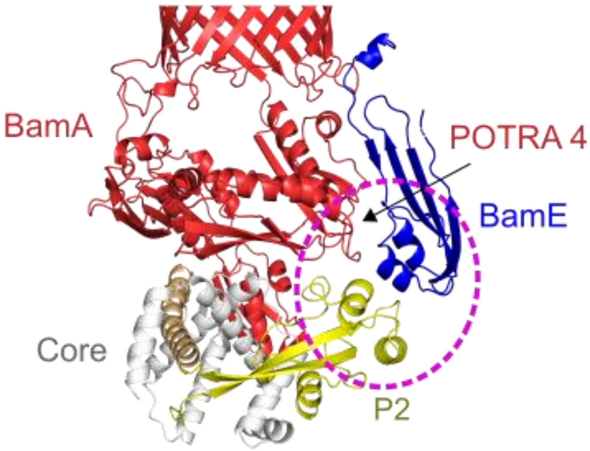

Figure S18. Views of interactions between different SurA domains and BAM subunits in an AlphaFold-Multimer generated model of the BAM-SurA complex. **(a)** The predicted interactions of the SurA core domain with BamA POTRA domains 1 and 2 and BamB (each ringed in magenta). Note, an interesting β-augmentation interaction is predicted between β2 of POTRA 1, and 5 residues close to the N-terminus of SurA. For clarity, BamC, BamD, BamE and the SurA P2 domain are not shown. **(b)** The predicted interaction of the SurA P2 domain with BamE and BamA POTRA domain 4 (ringed in magenta). For clarity, BamB, BamC, BamD and the SurA P1 domain are not shown.

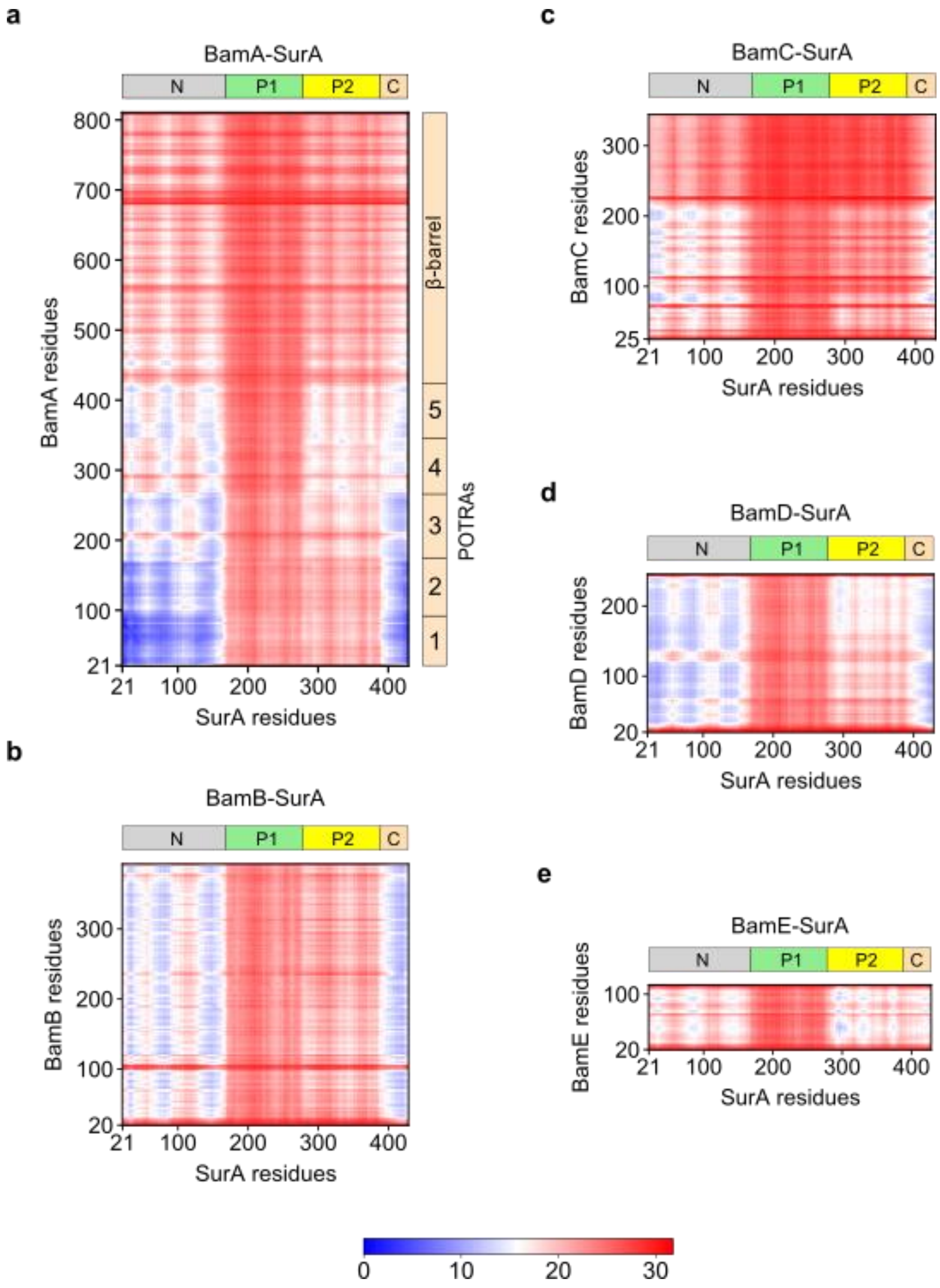

Figure S19. Predicted Alignment Errors (PAEs) between SurA and BAM components for an AlphaFold-Multimer generated model of the BAM-SurA complex. Plots PAEs between SurA and (**a**) BamA, (**b**) BamB, (**c**) BamC, (**d**) BamD, and (**e**) BamE. The PAE is a measure of the

expected error in position between pairs of residues (x,y) when residue x is aligned on residue y[5]. An overall low PAE score between pairs of residues in two domains (blue) indicates that AlphaFold predicts a well-defined relative position between the domains. Conversely, a high PAE score between pairs of residues in two domains (red) indicates uncertainly in the relative position of the domains[5].

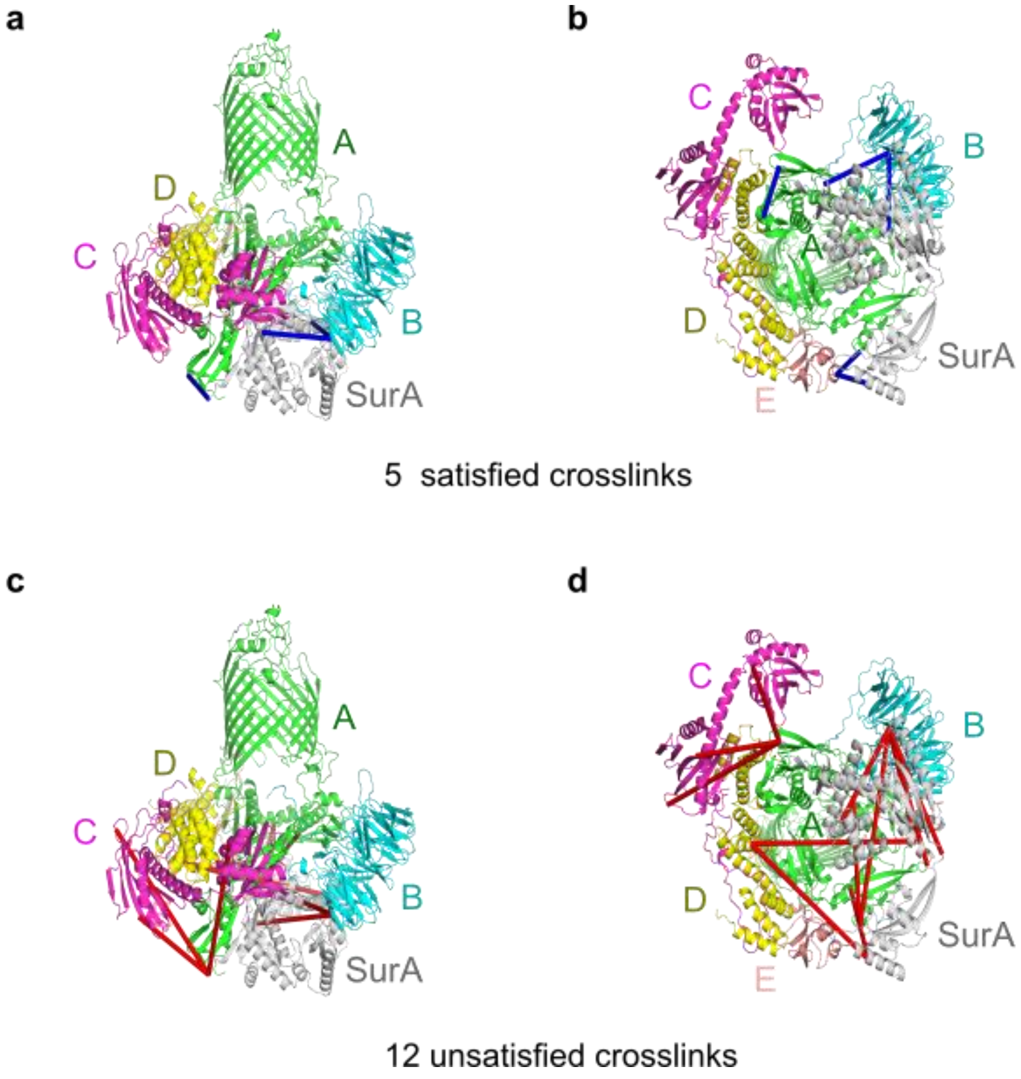

Figure S20. Experimental crosslinks between BAM and SurA are consistent with an AlphaFold generated model of the BAM-SurA complex. **(a,b)** Satisfied (blue lines) and **(c,d)** violated (red lines) cross-links mapped between SurA and BAM subunits in the AlphaFold BAM-SurA model. Side views and views from the periplasm are shown in (a,c) and (b,d), respectively. A satisfied crosslink is defined as one in which the $C\alpha$-$C\alpha$ distance between the residue pair is <30 Å[6]. The number of unsatisfied crosslinks suggests a dynamic complex, as it appears no single structure would be able to satisfy all experimentally measured cross-links. Details of the satisfied and unsatisfied crosslinks are given in **Table S5** and PyMOL session files generated using PyXlinkViewer[7] are provided as Source Data files.

Calculated M+H+	Calculated Mass	Deviation (ppm)	Peptide 1	Protein 1	From	To	Peptide2	Protein 2	From	To	Linkage position peptide 1	Linkage position peptide 2
2945.574	2945.573	0.25	YQISVKPQGYQQAVTVK	BamC	161	177	GAPQVVDK	SurA	20	28	K6	0
2305.219	2305.216	1.47	KEMIISEVR	SurA	135	143	SGQIMWKR	BamB	274	281	K1	K7
2340.196	2340.194	0.95	AAFSDFSKLVR	BamD	142	152	TDAAQKDR	SurA	390	397	K8	K6
2165.203	2165.200	1.43	LPAALEKVGMK	BamC	255	265	GAPQVVDK	SurA	20	28	K7	0
2301.240	2301.237	1.11	VKLEQIAADIK	SurA	306	316	MEDDIKK	BamA	304	310	K2	K6
3049.609	3049.607	0.55	HILLKPSPIMTDEQAR	SurA	290	305	SGQIMWKR	BamB	274	281	K5	K7
3020.588	3020.583	1.68	LLNLEQAGKPVADAASMQR	BamC	178	196	GAPQVVDK	SurA	20	28	K9	0
4579.412	4579.409	0.75	VVYRPDINQGNYLTANDVSKIR	BamE	26	47	HILLKPSPIMTDEQAR	SurA	290	305	K20	K5
2266.206	2266.201	2.09	ASAYVKILSN	SurA	420	430	SGQIMWKR	BamB	274	281	K6	K7
2927.396	2927.393	0.97	KFSEEAASWMQEQR	SurA	406	419	SGQIMWKR	BamB	274	281	K1	K7
2853.492	2853.489	0.83	AEGFVVKDIHFEGLQR	BamA	20	36	GAPQVVDK	SurA	20	28	K7	0
3958.142	3958.140	0.49	VVYRPDINQGNYLTANDVSKIR	BamE	26	47	VKLEQIAADIK	SurA	306	316	K20	K2
2663.478	2663.477	0.50	AAFSDFSKLVR	BamD	142	152	VKLEQIAADIK	SurA	306	316	K8	K2
3015.654	3015.645	3.15	IQELPGIFAQALSTAKK	SurA	237	253	SGQIMWKR	BamB	274	281	K16	K7
3837.839	3837.838	0.17	YSTEMMNVISAGLDKSATDAANAAQNR	BamC	197	223	GAPQVVDK	SurA	20	28	K15	0
2105.055	2105.056	-0.07	SGQIMWKR	BamB	274	281	TDAAQKDR	SurA	390	397	K7	K6
2102.095	2102.091	1.91	VGMKVTDSTR	BamC	262	271	GAPQVVDK	SurA	20	28	K4	0
2290.161	2290.161	0.24	NVDKTDAAQK	SurA	386	395	SGQIMWKR	BamB	274	281	K4	K7
2738.469	2738.466	1.03	HILLKPSPIMTDEQAR	SurA	290	305	YQKQK	BamA	214	218	K5	K3
2727.401	2727.400	0.60	GESKNISVTEVHAR	SurA	276	289	SGQIMWKR	BamB	274	281	K4	K7

Table S1. Detected cross-links between SurA and BAM subunits. Gene numbering is used for all proteins. A linkage position of 0 corresponds to the N-terminus of the peptide, as this corresponds to the N-terminus of the purified protein used for cross-linking studies.

Folding reaction	k_{obs} (x10^{-3} s^{-1})	Amplitude
BAM + SurA-WT	1.3 ± 0.08	0.9 ± 0.02
BAM + SurA-ΔP1	1.4 ± 0.06	0.9 ± 0.01
BAM + SurA-ΔP2	0.5 ± 0.02	0.8 ± 0.01
BAM + SurA-core	0.5 ± 0.01	0.9 ± 0.008
BAM alone	0.2 ± 0.06	0.2 ± 0.02
Empty liposomes	No folding	No folding
Empty liposomes + SurA-WT	No folding	No folding

Table S2. Observed rate constants for tOmpA folding into BAM-containing proteoliposomes composed of *E. coli* polar lipid extract in the presence or absence of different SurA domain deletion variants. For each folding condition two folding reactions were performed and averaged prior to fitting, each using an independently prepared batch of BAM proteoliposomes. Errors shown are the errors on a fit to a single exponential equation (see Methods).

Binding partners	Apparent K_d (μM)	Hill coefficient
tOmpA + SurA-WT	3.7 ± 1.0	1.2 ± 0.3
tOmpA + SurA-ΔP1	14.5 ± 9.3	1.0 ± 0.4
tOmpA + SurA-ΔP2	8.8 ± 2.8	0.7 ± 0.1
tOmpA + SurA-core	10.2 ± 2.3	1.4 ± 0.3

Table S3. Affinities for tOmpA for SurA-WT and domain deletion variants measured by MST. Samples contained 100 nM Alexa Fluor 488-labeled tOmpA, 100 μM-3 nM SurA or SurA domain deletion variant, 0.8 M urea, 20 mM Tris-HCl, pH 8.0, at 25 °C. Three replicates were performed and averaged prior to fitting. Errors shown are the errors on a fit to a Hill binding model (see Methods).

Binding partners	K_d (μM)
SurA-WT + BAM	0.6 ± 0.1
SurA-ΔP1 + BAM	0.6 ± 0.1
SurA-ΔP2 + BAM	1.2 ± 0.1
SurA-core + BAM	0.7 ± 0.1

Table S4. Affinities of SurA-WT and SurA domain deletion variants binding to BAM by MST. Samples contained 400 nM Alexa Fluor 488-labeled SurA variant, 25 μM-1 nM BAM complex in proteoliposomes composed of *E. coli* polar lipid extract, 150 mM NaCl, 20 mM Tris-HCl, pH 8.0, at 25 °C. Three replicates were performed and averaged prior to fitting. Errors shown are the errors on a fit to a 1:1 quadratic binding model (see Methods).

Crosslink type	SurA domain	SurA residue	BAM subunit	BAM residue	Distance (Å)
Satisfied	Core	A21 (N-term)	BamA	K27	17.1
	Core	K405	BamB	K280	26.1
	Core	K424	BamB	K280	28.3
	P2	K293	BamE	K45	14.9
	P2	K306	BamE	K45	15.3
(1) Violated (BamC)	Core	A21 (N-term)	BamC	K166	33.5
	Core	A21 (N-term)	BamC	K186	60.8
	Core	A21 (N-term)	BamC	K261	54.0
(2) Violated (Core)	Core	K134	BamB	K280	38.3
	Core	K394	BamB	K280	39.2
	Core	K394	BamD	K149	65.0
(3) Violated (P2)	P1-P2 linker	K278	BamB	K280	46.4
	P2	K293	BamA	K216	48.2
	P2	K306	BamA	K309	33.9
	P2	K388	BamB	K280	48.2
	P2	K293	BamB	K280	66.8
	P2	K306	BamD	K149	59.9

Table S5: Distances between residues involved in experimentally observed SurA-BAM crosslinks in the AlphaFold generated SurA-BAM model. Five observed DSBU crosslinks are satisfied in the AlphaFold model. We define a crosslink as being satisfied if there is a straight line distance between the Cα atoms of the residues involved of <30 Å[6]. The satisfied crosslinks are in agreement with the major interaction sites between SurA and BamA, BamB and BamE detected by HDX-MS (**Fig. 2**). The 12 violated crosslinks in the model can be separated into three types as indicated: (1) from BamC to the SurA core domain, likely due to dynamics of the helix-grip domains of BamC[1], (2) from the SurA core domain to BamB and BamD, suggestive of dynamic movements of the core domain while bound to POTRAs 1 and 2, and (3) P2 and the P1-linker to various locations in BamA, BamB, and BamD. The binding data in Figure S5 suggests that the main contribution to affinity for BAM comes from the SurA core domain. Therefore, it is possible that these unsatisfied crosslinks could be explained by the P2 domain leaving and rebinding its binding site to BamE/POTRA 4 while SurA remains tethered to BAM via its core domain. PyMOL session files of the BAM-SurA AlphaFold model with the satisfied and unsatisfied crosslinks indicated are provided as Source Data files.

Protein	Residue	Group	Protein	Region	Residue	Group	Distance (Å)
BamA	R79	N	SurA	Core	Q23	O	2.75
BamA	V77	N	SurA	Core	D26	O	3.29
BamA	S60	OG	SurA	Core	D26	OD1	2.89
BamA	S60	OG	SurA	Core	S40	OG	3.73
BamA	R64	NH2	SurA	Core	D41	OD2	3.61
BamA	R79	O	SurA	Core	Q23	N	3
BamA	V77	O	SurA	Core	V25	N	2.99
BamA	V77	O	SurA	Core	D26	N	3.58
BamA	V75	O	SurA	Core	V28	N	3.53
BamA	G275	O	SurA	P2	N336	ND2	2.37
BamA	E73	OE1	SurA	Core	K424	NZ	2.36
BamB	N186	ND2	SurA	Core-P1 linker	E156	OE1	3.81
BamB	R215	NH1	SurA	Core	E408	OE1	3.81
BamB	M226	O	SurA	P1	Q245	NE2	3.74
BamB	I277	O	SurA	P1	R260	NH2	3.1
BamE	Q54	NE	SurA	P2	P296	O	2.26
BamE	R47	NH2	SurA	P2	I297	O	3.7

Table S6: Hydrogen bonds in the AlphaFold predicted BAM-SurA complex. Interactions were analysed using PDBePISA[8].

Protein	Residue	Group	Protein	Region	Residue	Group	Distance
BamA	R64	NH2	SurA	Core	D41	OD1	3.94
BamA	R64	NH2	SurA	Core	D41	OD2	3.61
BamA	E73	OE1	SurA	Core	K424	NZ	2.36
BamB	R215	NH1	SurA	Core	E408	OE1	3.81
BamB	R215	NH2	SurA	Core	E408	OE1	3.66
BamB	R215	NH1	SurA	Core	E408	OE2	3.86
BamB	R215	NH2	SurA	Core	E408	OE2	3.51
BamB	R243	NH1	SurA	Core	E416	OE1	3.26
BamB	R243	NH1	SurA	Core	E416	OE2	3.25

Table S7: Salt bridges in the AlphaFold predicted BAM-SurA complex. Interactions were analysed using PDBePISA[8].

Data Set	BAM ± SurA	BAM ± SurA-core	SurA ± BAM
**HDX reaction details**	10 mM potassium phosphate, pD 8.0, 0.02 % DDM, 4 ºC	10 mM potassium phosphate, pD 8.0, 0.02 % DDM, 4 ºC	10 mM potassium phosphate, pD 8.0, 0.02 % DDM, 4 ºC
**HDX time course (min)**	0.5, 2, 30, 120 min		
**HDX control samples**	Maximally-labeled controls were not performed		
**Back-exchange**	~ 30 %		
**# of Peptides**	BamA 190<br>BamB 46<br>BamC 64<br>BamD 62<br>BamE 20	BamA 190<br>BamB 46<br>BamC 64<br>BamD 63<br>BamE 20	SurA 64
**Sequence coverage**	BamA 81.27 %<br>BamB 70.51 %<br>BamC 93.75 %<br>BamD 86.73 %<br>BamE 99.04 %	BamA 81.27 %<br>BamB 70.51 %<br>BamC 93.75 %<br>BamD 86.73 %<br>BamE 99.04 %	SurA 83.86 %
**Average peptide length / Redundancy**	BamA 10.76 / 3.19<br>BamB 10.41 / 1.82<br>BamC 12.05 / 2.57<br>BamD 11.23 / 3.55<br>BamE 11.90 / 2.31	BamA 10.76 / 3.19<br>BamB 10.41 / 1.82<br>BamC 12.05 / 2.57<br>BamD 11.24 / 3.61<br>BamE 11.90 / 2.31	11.73 / 2.19
**Replicates (biological or technical)**	3 (technical)	3 (technical)	3 (technical)
**Repeatability (average SD)**	Bam A 0.0312<br>BamB 0.0388<br>BamC 0.0320<br>BamD 0.0277<br>BamE 0.0552	Bam A 0.0305<br>BamB 0.0365<br>BamC 0.0296<br>BamD 0.0262<br>BamE 0.0381	0.0456

**Significant differences in HDX**<br>**(Δ HDX > X Da)**	Bam A 0.59 Da<br>BamB 0.55 Da<br>BamC 0.58 Da<br>BamD 0.54 Da<br>BamE 0.73 Da<br>(99 % CI) in summed data	Bam A 0.57 Da<br>BamB 0.37 Da<br>BamC 0.58 Da<br>BamD 0.51 Da<br>BamE 0.73 Da<br>(99 % CI) in summed data	SurA 0.83 Da<br>(99 % CI) in summed data

Table S8: HDX Data Summary Table. SD = standard deviation, CI = confidence interval.

Supplementary Information References

1. Iadanza, M.G. et al. Lateral opening in the intact β-barrel assembly machinery captured by cryo-EM. *Nat Commun* **7**, 12865; 10.1038/ncomms12865 (2016).
2. Lau, A.M.C., Ahdash, Z., Martens, C. & Politis, A. Deuteros: software for rapid analysis and visualization of data from differential hydrogen deuterium exchange-mass spectrometry. *Bioinformatics* **35**, 3171-3173 (2019).
3. Humes, J.R. et al. The Role of SurA PPIase Domains in Preventing Aggregation of the Outer-Membrane Proteins tOmpA and OmpT. *J Mol Biol* **431**, 1267-1283 (2019).
4. White, P. et al. The role of membrane destabilisation and protein dynamics in BAM catalysed OMP folding. *Nat Commun* **12**, 4174; 10.1038/s41467-021-24432-x (2021).
5. Evans, R. et al. Protein complex prediction with AlphaFold-Multimer. *bioRxiv*, 2021.10.04.463034 (2021).
6. Merkley, E.D. et al. Distance restraints from crosslinking mass spectrometry: mining a molecular dynamics simulation database to evaluate lysine-lysine distances. *Protein Sci* **23**, 747-59 (2014).
7. Schiffrin, B., Radford, S.E., Brockwell, D.J. & Calabrese, A.N. PyXlinkViewer: A flexible tool for visualization of protein chemical crosslinking data within the PyMOL molecular graphics system. *Protein Sci* **29**, 1851-1857 (2020).
8. Krissinel, E. & Henrick, K. Inference of macromolecular assemblies from crystalline state. *J Mol Biol* **372**, 774-97 (2007).
