# Supplementary material for: Dynamic interplay between the periplasmic chaperone SurA and the BAM complex in outer membrane protein folding
Source: Commun Biol. 2022 Jun 8;5:560. doi: 10.1038/s42003-022-03502-w (PMC9177699; doi:10.1038/s42003-022-03502-w)
Supplement: Supplementary file 2 — Description of additional Supplementary Files [file 42003_2022_3502_MOESM2_ESM.pdf]

Supplementary Videos

Supplementary Video 1. Regions of BAM which exhibit HDX protection in the presence of SurA-WT. HDX protection (blue) in the BamA β-barrel and the three major regions in the BAM periplasmic ring (POTRAs 1-2, BamB, and BamE) are highlighted. Regions in white show no change in deuterium uptake in the presence of SurA, while those in dark grey denote sequences for which peptides were not detected. HDX-MS results are mapped onto a crystal structure of the BAM complex in a 'lateral-closed' ('inward-open') state (PDB: 5AYW [12]). A PyMOL session file of the movie is included as a supplementary data file (**Supplementary Data 1**).

Supplementary Video 2. HDX-MS results for SurA-WT in the presence of BAM**.** Regions of SurA-WT which exhibit HDX protection or deprotection in the presence of BAM are highlighted in blue or red, respectively. Regions in white show no change in deuterium uptake in the presence of SurA, while those in dark grey denote sequences for which peptides were not detected. HDX-MS results are mapped onto the crystal structure of SurA-WT (PDB: 1M5Y [31]) PyMOL session file of the movie is included as a supplementary data file (**Supplementary Data 2**).

Supplementary Video 3. Comparison of an AlphaFold-Multimer generated model of the BAM-SurA complex with HDX-MS results. HDX protection (blue) in the BamA β-barrel and the three major regions in the BAM periplasmic ring (POTRAs 1-2, BamB, and BamE) are highlighted. Regions in white show no change in deuterium uptake in the presence of SurA, while those in dark grey denote sequences for which peptides were not detected. The SurA core, P1 and P2 domains are coloured in orange, green and yellow, respectively. A PyMOL session file of the movie is included as a supplementary data file (**Supplementary Data 4**).

Supplementary Data

Supplementary Data 1. PyMOL session file used to make Supplementary Video 1 showing regions of HDX protection in BAM in the presence of SurA-WT**.**

Supplementary Data 2. PyMOL session file used to make Supplementary Video 2 showing regions of HDX protection in SurA-WT in the presence of BAM**.**

Supplementary Data 3. PDB file of an AlphaFold-Multimer generated model of the BAM-SurA complex.

Supplementary Data 4. PyMOL session file used to make Supplementary Video 3 showing a comparison of an AlphaFold-Multimer generated model of the BAM-SurA complex with HDX results for BAM in the presence of SurA-WT**.**

Supplementary Data 5. Reporting summary for XL-MS data on the BAM-SurA complex.

Supplementary Data 6. PyMOL session file of the AlphaFold-Multimer generated model of the BAM-SurA complex with satisfied crosslinks highlighted in blue using PyXlinkViewer [119].

Supplementary Data 7. PyMOL session file of the AlphaFold-Multimer generated model of the BAM-SurA complex with unsatisfied crosslinks highlighted in red using PyXlinkViewer [119].

Supplementary Data 8. Source data for MST experiments shown in Fig. 1, Fig. S7, Fig. S13 and Fig. S15.

Supplementary Data 9. Source data for deuterium uptake plots shown in Fig. 2, Fig. 3 and Fig. S9

Supplementary Data 10. Source data for tOmpA folding experiments shown in Fig. 4, Fig. S11 and Fig. S12.

Supplementary Data 11. Source data for nephelometry experiments shown in Fig. S14.
